# The FDA-approved excipient N,N-dimethylacetamide improves survival and attenuates inflammatory pathways in a murine model of endotoxemia

**DOI:** 10.1016/j.biopha.2026.119403

**Published:** 2026-04-18

**Authors:** Zhihui Xiao, Sean Carrig, Sandra E. Reznik

**Affiliations:** a Department of Medicine, Perelman School of Medicine, University of Pennsylvania, Philadelphia, PA, United States; b Children’s Hospital of Philadelphia, University of Pennsylvania, Philadelphia, PA, United States; c Department of Pharmaceutical Sciences, St. John’s University, Jamaica, NY, United States; d Departments of Pathology and Obstetrics and Gynecology and Women’s Health, Albert Einstein College of Medicine, Bronx, NY, United States

**Keywords:** Sepsis, Endotoxemia, N, N-dimethylacetamide (DMA), Inflammation, Cytokines, Lipopolysaccharide

## Abstract

Sepsis is a life-threatening organ dysfunction resulting from a dysregulated host response to infection and remains a major public health burden. According to the Centers for Disease Control and Prevention, approximately 1.7 million adults in the United States develop sepsis annually. We previously demonstrated that the United States Food and Drug Administration–approved drug excipient *N,N*-dimethylacetamide (DMA) suppresses inflammatory responses through inhibition of the nuclear factor kappa B (NF-κB) pathway. In the present study, DMA completely prevented mortality in a murine model of acute (24 h) moderate endotoxemia and significantly improved survival in acute severe endotoxemia. In mice with severe endotoxemia, serum interleukin-6 (IL-6) and tumor necrosis factor–α (TNF-α) levels were significantly reduced in DMA-treated animals compared with lipopolysaccharide (LPS)-only controls. In long-term (96 h) moderate endotoxemia, pretreatment with DMA significantly improved clinical parameters, including mobility, heart rate, and respiratory rate. In the liver, DMA suppressed LPS-induced expression of pro-inflammatory cytokines and acute-phase proteins, enhanced expression of the anti-inflammatory cytokine IL-10, attenuated NLRP3 inflammasome activation, and modulated leukocyte infiltration in pretreated mice. In U937 macrophages, DMA inhibited LPS-induced release of IL-6 and TNF-α. Notably, macrophages differentiated in the presence of DMA exhibited an attenuated inflammatory response to subsequent LPS stimulation. Collectively, these findings identify DMA as a promising adjunctive therapeutic anti-inflammatory candidate in endotoxemia that warrants further evaluation in clinically relevant models of sepsis.

## 1. Introduction

Sepsis is a life-threatening syndrome characterized by a dysregulated host response to infection, resulting in life-threatening organ dysfunction [1]. Despite advances in critical care, sepsis remains a leading cause of morbidity and mortality worldwide. In the United States alone, an estimated 1.7 million adults develop sepsis each year, and approximately 350,000 die during hospitalization or are discharged to hospice care [2]. Septic shock, defined by vasopressor-dependent hypotension and elevated lactate levels despite adequate fluid resuscitation, is associated with particularly high mortality [1]. Current treatment strategies remain largely supportive—centered on infection control, hemodynamic stabilization, and organ support—while no approved pharmacologic therapy directly targets the dysregulated inflammatory cascade that drives sepsis pathogenesis [3,4]. Numerous anti-cytokine, antioxidant, and immunomodulatory approaches have failed to yield meaningful clinical benefit, underscoring the urgent need for safe, mechanism-based therapeutic agents [5,6].

N,N-dimethylacetamide (DMA) is an FDA-approved excipient widely used as a solvent in clinical drug formulations, including busulfan and teniposide [7–9]. Although long regarded as pharmacologically inert, we and others identified DMA as a previously unrecognized small-molecule inhibitor of nuclear factor κB (NF-κB) signaling with potent anti-inflammatory and immunomodulatory activity [10–12]. Our prior work demonstrated that DMA suppresses LPS- and TNFα-induced production of proinflammatory mediators—including IL-6, TNFα, CCL2, and HMGB1—attenuates oxidative and nitrosative stress and reduces leukocyte infiltration and tissue injury in diverse inflammatory settings [10,12,13]. DMA also alleviates preterm birth–associated inflammation, mitigates colitis, and diminishes neuroinflammation by suppressing NF-κB–dependent cytokine expression and amyloid β precursor protein production in microglial cells [10,13–16]. These findings establish DMA as a clinically accessible small molecule with broad anti-inflammatory efficacy and a well-characterized safety profile.

Given the central role of NF-κB activation in sepsis pathophysiology and the established anti-inflammatory actions of DMA, we hypothesized that DMA could confer therapeutic protection in sepsis by suppressing NF-κB–driven inflammatory signaling. Here, we report that DMA treatment significantly improves survival in LPS-induced murine endotoxemia, accompanied by a marked reduction in circulating proinflammatory cytokines, attenuation of hepatic injury, and preservation of tissue architecture. These findings provide compelling preclinical evidence that repurposing DMA—an FDA-approved excipient with newly discovered NF-κB–inhibitory activity—represents a promising and translationally feasible strategy for the treatment of sepsis and related inflammatory diseases.

## 2. Methods

### 2.1 . In-vivo protocols

All experimental protocols were approved by the St. John’s University Institutional Animal Care and Use Committee (IACUC). All methods were carried out in accordance with relevant guidelines and regulations. All methods are reported in accordance with ARRIVE guidelines (https://arriveguidelines.org). Two doses of LPS (30 mg/kg and 60 mg/kg) were selected to model moderate and severe endotoxemia, respectively, based on prior studies demonstrating dose-dependent increases in systemic inflammation and mortality in murine models.

#### 2.1.1. Acute endotoxemia

Male C57BL/6 mice were administered either normal saline (Teknova) or N,N-dimethylacetamide (DMA; 1.57 g/kg) intraperitoneally (ip) 20 min prior to endotoxemia induction (T–20). At T0, mice received an ip injection of either normal saline or lipopolysaccharide (LPS; 60 mg/kg; *Escherichia coli* O26:B6, Sigma-Aldrich) to induce severe endotoxemia. Experimental endpoints were 1.5, 4, 8, 12, and 24 h post-endotoxemia. At each time point, mice were randomly assigned to one of four groups (n = 6 per group): (1) sham, receiving normal saline at T–20 min and T0; (2) DMA control, receiving DMA at T–20 min and saline at T0; (3) severe endotoxemia, receiving LPS at T0; and (4) DMA-treated severe endotoxemia, receiving DMA at T–20 min followed by LPS at T0. Mice in the 24-h endpoint groups received subcutaneous fluid resuscitation with 0.5 ml of 5% dextrose at T12h. At each endpoint, mice were anesthetized with isoflurane, and blood was collected by terminal cardiac puncture. Animals were then euthanized by cervical dislocation, and the heart, lungs, spleen, kidneys, and liver were harvested for subsequent analyses.

#### 2.1.2. Prolonged endotoxemia

Male C57BL/6 mice were administered either normal saline or DMA (1.57 g/kg) ip 20 min prior to endotoxemia induction (T–20 min). At T0, mice received an ip injection of either normal saline or LPS at 30 mg/kg or 60 mg/kg to induce moderate or severe endotoxemia, respectively. Experimental endpoints for prolonged endotoxemia were 48, 72, and 96 h post-endotoxemia. At each endpoint, mice were assigned to one of six groups: (1) sham, receiving normal saline at T–20 min and T0 (n = 4); (2) DMA control, receiving DMA at T–20 and saline at T0 (n = 4); (3) moderate endotoxemia, receiving 30 mg/kg LPS at T0 (n = 6); (4) DMA-treated moderate endotoxemia, receiving DMA at T–20 min followed by 30 mg/kg LPS at T0 (n = 6); (5) severe endotoxemia, receiving 60 mg/kg LPS at T0 (n = 6); and (6) DMA-treated severe endotoxemia, receiving DMA at T–20 min followed by 60 mg/kg LPS at T0 (n = 6). All mice received subcutaneous fluid resuscitation with 0.5 ml of 5% dextrose every 12 h throughout the study. Mice in the DMA control and DMA-treated moderate and severe endotoxemia groups received additional DMA dosing (1.57 g/kg, intraperitoneally) every 24 h. At each endpoint, mice were anesthetized with isoflurane, and blood was collected by terminal cardiac puncture. Animals were then euthanized by cervical dislocation, and the heart, lungs, spleen, kidneys, and liver were harvested for subsequent organ damage analyses.

#### 2.1.3. Vital sign monitoring using MouseOx

Vital signs of mice subjected to 96-h moderate endotoxemia were continuously assessed using a MouseOx pulse oximeter (STARR Life Sciences). Recorded parameters included motion, heart rate (HR), breath rate (BrR), and arterial oxygen saturation (SpO_2_). Mice used in this study were 18–22 weeks of age and weighed >26 g at the time of experimentation. Prior to initiation of the 96-h study, mice were acclimated to the MouseOx neck collar through 20-min training sessions, with a minimum of 20 sessions per animal (one daytime and one nighttime session per day). Following completion of training, mice were administered either normal saline or DMA (1.57 g/kg) ip 20 min prior to endotoxemia induction (T–20 min). At T0, mice received an ip injection of either normal saline or LPS (30 mg/kg) to induce moderate endotoxemia. The experimental endpoint was 96 h post-endotoxemia. Mice were assigned to one of four groups: (1) sham, receiving normal saline at T–20 min and T0 (n = 4); (2) DMA control, receiving DMA at T–20 min and saline at T0 (n = 4); (3) moderate endotoxemia, receiving 30 mg/kg LPS at T0 (n = 6); and (4) DMA-treated moderate endotoxemia, receiving DMA at T–20 min followed by 30 mg/kg LPS at T0 (n = 6). All mice received subcutaneous fluid resuscitation with 0.5 ml of 5% dextrose every 12 h throughout the study. Mice in the DMA control and DMA-treated moderate endotoxemia groups received additional DMA dosing (1.57 g/kg, ip) every 24 h. Vital signs were recorded for each mouse during one daytime and one nighttime session (15–30 min per session) at each time point (T0, T12, T24, T36, T48, T60, T72, T84, and T96). Data were acquired at a sampling frequency of 1 Hz (one recording per second).

MouseOx data were exported and processed using Microsoft Excel. Calculation formulas are described below.

- Motion:
Mobility(activity/min)=Sumofactivity/Totalrecordingtime(sec)×60(sec)
$${\text{DMobility}}_{\left(T\right)}={\;\text{Avg}\;\text{Mobility}}_{\left(T\right)}-{\;\text{Avg}\;\text{Mobility}}_{\left(T0\right)}$$
- HR:
AvgHR(T)=Sumoferror−freeHR/Totalnumberoferror−freerecording
$${DHR}_{\left(T\right)}=Avg\;{HR}_{\left(T\right)}-Avg\;{HR}_{\left(TO\right)}$$
- BrR:
AvgBrR(T)=Sumoferror−freeBrR/Totalnumberoferror−freerecording
$${DBrR}_{\left(T\right)}=Avg\;{BrR}_{\left(T\right)}-Avg\;{BrR}_{\left(TO\right)}$$
- O_2_:
AvgO2(T)=Sumoferror−freeO2/Totalnumberoferror−freerecording

At each endpoint, mice were anesthetized with isoflurane, and blood was collected by terminal cardiac puncture. Animals were then euthanized by cervical dislocation, and the heart, lungs, spleen, kidneys, and liver were harvested for subsequent organ damage analyses.

#### 2.1.4. Survival studies

##### 2.1.4.1. Acute endotoxemia survival.

Twelve- and 24–h survival data were pooled from animals subjected to 24-, 48-, 72-, and 96-h endotoxemia protocols. The resulting sample sizes were *n* = 24 for moderate endotoxemia and *n* = 18 for severe endotoxemia.

##### 2.1.4.2. Long-term endotoxemia survival.

Survival was monitored over a 96-h period in mice subjected to prolonged endotoxemia and used for long-term survival analyses.

#### 2.1.5. Tissue collection for protein, RNA, and histological analyses

For protein analysis, organ samples were snap-frozen in liquid nitrogen immediately after collection and stored at −80 °C until use. For RNA analysis, tissues were diced into pieces ≤ 5 mm in greatest dimension and transferred to microcentrifuge tubes containing RNAlater solution (Invitrogen). Samples were incubated overnight at 4 °C to allow complete tissue penetration and RNA stabilization. The following day, RNAlater was removed, and tissues were stored at −80 °C. For histological analyses, organ samples were fixed in 4% paraformaldehyde in phosphate-buffered saline (PBS). After fixation for 24 h at room temperature, the paraformaldehyde solution was removed, and tissues were transferred to 75% ethanol for storage until processing.

#### 2.1.6. Protein analysis

##### 2.1.6.1. Liver tissue protein collection.

Snap-frozen liver tissue samples were thawed on ice and homogenized in RIPA buffer (G-Biosciences) supplemented with phosphatase and protease inhibitors (Thermo Scientific). Samples were sonicated on ice at 25% amplitude using four cycles of 3 s on and 2 s off (Thermo Scientific). Homogenates were then incubated on ice for 30 min and centrifuged at 12,000 rpm for 20 min at 4 °C. The resulting supernatants were collected and aliquoted into three sets for independent bicinchoninic acid (BCA) protein quantification and Western blot analyses, then stored at −80 °C until further use.

##### 2.1.6.2. BCA assay.

Total protein concentrations of liver samples were determined using a bicinchoninic acid (BCA) protein assay kit (Pierce, Thermo Scientific) according to the manufacturer’s instructions. Absorbance was measured at 562 nm using a Synergy H1 microplate reader. Absorbance values were exported to Microsoft Excel and corrected by subtraction of blank well readings. For each assay, a standard curve was generated using bovine serum albumin (BSA) standards, with BSA concentration plotted on the x-axis and corrected absorbance on the y-axis. Sample protein concentrations were calculated by interpolation from the standard curve.

##### 2.1.6.3. Western blotting.

Protein samples for western blotting were prepared by diluting 30 μg of total protein in deionized water containing 1 × Laemmli sample buffer (Bio-Rad) and 10% β-mercaptoethanol (BME). Samples were resolved on 4–20% Tris-glycine gradient gels (Invitrogen, Novex WedgeWell) and electrophoresed at 65 V for 4 h in Tris-glycine SDS running buffer (Invitrogen, Novex). Proteins were transferred to polyvinylidene difluoride (PVDF) membranes (Immobilon, 0.45 μm pore size) at 200 mA for 2 h. Membranes were blocked in 5% bovine serum albumin (BSA) prepared in Tris-buffered saline containing 0.1% Tween-20 (TBST) for 1 h at room temperature, followed by incubation with up to two primary antibodies overnight at 4 °C. Antibodies are listed in Table S1. Membranes were then washed three times with TBST and incubated with horseradish peroxidase (HRP)-conjugated goat anti-rabbit IgG secondary antibody (Invitrogen) for 1 h at room temperature. After three additional washes with TBST, immunoreactive bands were detected using enhanced chemiluminescence (ECL) substrate (Pierce, Thermo Scientific) and imaged with a ChemiDoc MP imaging system (Bio-Rad). For reprobing, membranes were stripped using Restore stripping buffer (Thermo Scientific) for 1 h, washed three times with TBST, and reblocked in 5% BSA/TBST for 1 h prior to incubation with additional primary antibodies. Protein band intensities were quantified using Image Lab software (Bio-Rad) with background subtraction applied uniformly across blots. Adjusted intensity values were exported to Microsoft Excel for analysis. Relative protein expression was calculated by normalizing target protein band intensities to the corresponding GAPDH band intensity. BCA assays and Western blot analyses of mouse liver tissue were performed independently in triplicate for each time point (three sets of aliquots). Normalized values from independent experiments were pooled and analyzed using Prism software.

#### 2.1.7. Gene expression analysis

##### 2.1.7.1. RNA isolation.

Frozen tissue samples were thawed on ice, and total RNA was extracted using TRIzol reagent (Invitrogen) according to the manufacturer’s protocol. RNA pellets were resuspended in DNase/RNase-free water (Invitrogen) and aliquoted into three sets for independent downstream analyses. Total RNA concentration and purity were assessed using a NanoDrop spectrophotometer (Thermo Scientific).

##### 2.1.7.2. Genomic DNA digestion and RNA purification.

Genomic DNA contamination in extracted RNA samples was removed using RQ1 RNase-Free DNase (Promega) according to the manufacturer’s instructions. RNA was subsequently purified by precipitation with 0.5 M ammonium acetate (Fisher Chemical) and isopropanol. Purified RNA pellets were air-dried and resuspended in DNase/RNase-free water. RNA concentration and purity were then assessed using a NanoDrop spectrophotometer (Thermo Scientific).

##### 2.1.7.3. cDNA synthesis and quantitative PCR (qPCR).

Complementary DNA (cDNA) was synthesized from purified RNA using a RevertAid First Strand cDNA Synthesis Kit (Thermo Scientific) according to the manufacturer’s instructions. For liver samples collected at each endotoxemia time point, three independent sets of cDNA were generated from separately purified RNA samples. Target gene expression was quantified by real-time PCR (qPCR) using SYBR Green master mix (Applied Biosystems) on a QuantStudio 3 instrument (Applied Biosystems, Thermo Fisher Scientific). Primer sequences are listed in Supp. Table 2. qPCR data were exported to Microsoft Excel for calculation. Each cDNA set was assayed in duplicate, and qPCR assays were repeated once per set (three independent cDNA sets total). Data from all three sets were pooled, then graphed and analyzed using GraphPad Prism.

#### 2.1.8. Histology

The top lobe of the liver was used for histological analysis. Three tissue sections were obtained from the left, middle, and right regions of the lobe and stained with hematoxylin and eosin (H&E). For each slide, one image was captured from the top, center, and bottom of the section under a 10 × objective using a Nikon Ts2R-FL inverted microscope by a researcher blinded to the experimental groups. Images were evaluated by two trained, blinded researchers using a Modified Hepatic Activity Index. Scores from the two evaluators were averaged and analyzed in GraphPad Prism for statistical comparisons.

### 2.2. In-vitro protocols

#### 2.2.1. Cell culture

Murine bone marrow (BM) neutrophils were isolated from Swiss Webster mice (Taconic) following the protocol of Swamydas et al. [17] and cultured in RPMI 1640 medium (Corning) supplemented with 10% fetal bovine serum (FBS) and 1% penicillin/streptomycin.

Human U937 promonocytic cells were maintained in RPMI 1640 medium (Corning) supplemented with 10% FBS and 1% penicillin/streptomycin.

#### 2.2.2. Viability assays

##### 2.2.2.1. XTT assay.

Human U937 promonocytic cells and murine bone marrow (BM)–derived neutrophils were seeded into 96-well flat-bottom plates. DMA was added at final concentrations of 0.1, 1, 2.5, 5, 10, 25, 50, 75, or 100 mM. U937 cells were incubated with DMA for 72, 96, or 120 h, whereas BM-derived neutrophils were incubated for 24 or 48 h. At the end of each treatment period, freshly prepared XTT reagent [2,3-bis(2-methoxy-4-nitro-5-sulfophenyl)-2H-tetrazolium-5-carboxanilide; Biotium] containing 1 mg/ml XTT and 7.5 μg/ml phenazine methosulfate (ACROS Organics) was added to each well and incubated for 30–60 min at 37 °C. Absorbance was measured at 450 nm using a Synergy H1 microplate reader (BioTek).

Data were exported to Microsoft Excel, and cell viability was calculated by normalizing absorbance values to those of untreated control cells. Each experiment was performed in triplicate and independently repeated three times. Pooled data were analyzed using GraphPad Prism.

##### 2.2.2.2. Trypan blue exclusion assay.

U937 cells were seeded into 6-well plates and treated with DMA at final concentrations of 0.1, 1, or 10 mM for 72, 96, or 120 h. Control cells received RPMI complete medium alone. At each time point, cells were harvested and mixed with 0.4% trypan blue solution (Gibco), and viable cells were counted using a hemocytometer under light microscopy. Cell density values were exported to Microsoft Excel, and cell viability was calculated by normalizing each treatment group to the corresponding control group. Each experiment was performed in triplicate and independently repeated three times. Pooled data were analyzed using GraphPad Prism.

#### 2.2.3. Inflammation studies

##### 2.2.3.1. U937 macrophage assay.

U937 cells were seeded into 96-well plates and differentiated into macrophage-like cells using phorbol 12-myristate 13-acetate (PMA; 10 ng/ml; Sigma-Aldrich) in the presence or absence of DMA (10 mM). After 72 h, adherent cells (U937-derived macrophages) were washed once with Dulbecco’s phosphate-buffered saline (DPBS) and maintained in RPMI medium. U937-derived macrophages were then stimulated with LPS (1 μg/ml) in the absence or presence of DMA (0.1, 1, or 10 mM) for 24 or 48 h. Control cells received culture medium alone. Following incubation, culture supernatants were collected and stored at −80 °C for subsequent analyses. Human IL-6 and TNFα levels were quantified by ELISA as described below. Each experiment was performed in triplicate and independently repeated three times. Pooled data were analyzed using GraphPad Prism.

##### 2.2.3.2. Bone marrow leukocyte assay.

Bone marrow–derived neutrophils were seeded into 96-well plates and treated with LPS (1 μg/ml) in the presence or absence of DMA (0.1, 1, or 10 mM) for 24 or 48 h. Control cells received RPMI complete medium alone. Following treatment, culture supernatants were collected and stored at −80 °C for subsequent analysis. Levels of murine IL-6 and TNFα were quantified using ELISA as described below. Each experiment was performed in triplicate and independently repeated three times. Pooled data were analyzed using GraphPad Prism.

##### 2.2.3.3. Enzyme-linked immunoassays (ELISAs)

###### 2.2.3.3.1. Murine cytokine ELISAs (mIL-6 and mTNFα).

Murine IL-6 (mIL-6; Invitrogen, 88–7064) and TNFα (mTNFα; Invitrogen, 88–7324) levels in bone marrow neutrophil culture supernatants and mouse serum were quantified using commercial ELISA kits according to the manufacturer’s instructions. High-binding 96-well plates (Corning) were coated with capture antibodies, and assays were performed following the supplied protocols. Samples were diluted in 1 × ELISA/ELISPOT Diluent to ensure values fell within the linear range of the standard curve. Absorbance was measured at 450 nm with wavelength correction at 650 nm using a Synergy H1 microplate reader. Data were exported to Microsoft Excel, and absorbance values were normalized by subtraction of blank readings. Standard curves were generated by plotting cytokine concentration on the x-axis against normalized absorbance on the y-axis. Cytokine concentrations in samples were calculated by interpolation from the standard curves. For mouse serum samples, ELISA measurements were repeated at least once or until sample depletion. Final cytokine values were graphed and analyzed using GraphPad Prism.

###### 2.2.3.3.2 . Human cytokine ELISAs (hIL-6 and hTNFα).

Human IL-6 (hIL-6; Invitrogen, CHC1263) and TNFα (hTNFα; Invitrogen) levels in U937-derived macrophage culture supernatants were quantified using commercial ELISA kits according to the manufacturer’s instructions. High-binding 96-well plates (Corning) were coated with capture antibodies, and assays were performed following the supplied protocols. Samples were diluted in assay buffer to ensure measurements fell within the linear range of the standard curve. Absorbance was measured at 450 nm with wavelength correction at 650 nm using a Synergy H1 microplate reader. Data were exported to Microsoft Excel, and absorbance values were normalized by subtraction of blank readings. Standard curves were generated by plotting cytokine concentration on the x-axis against normalized absorbance on the y-axis. Cytokine concentrations were calculated by interpolation from the standard curves. Final cytokine values were graphed and analyzed using GraphPad Prism.

## 3. Results

### 3.1. DMA pretreatment improves survival and stabilizes vital signs during acute and prolonged endotoxemia

**Fig. 1.**
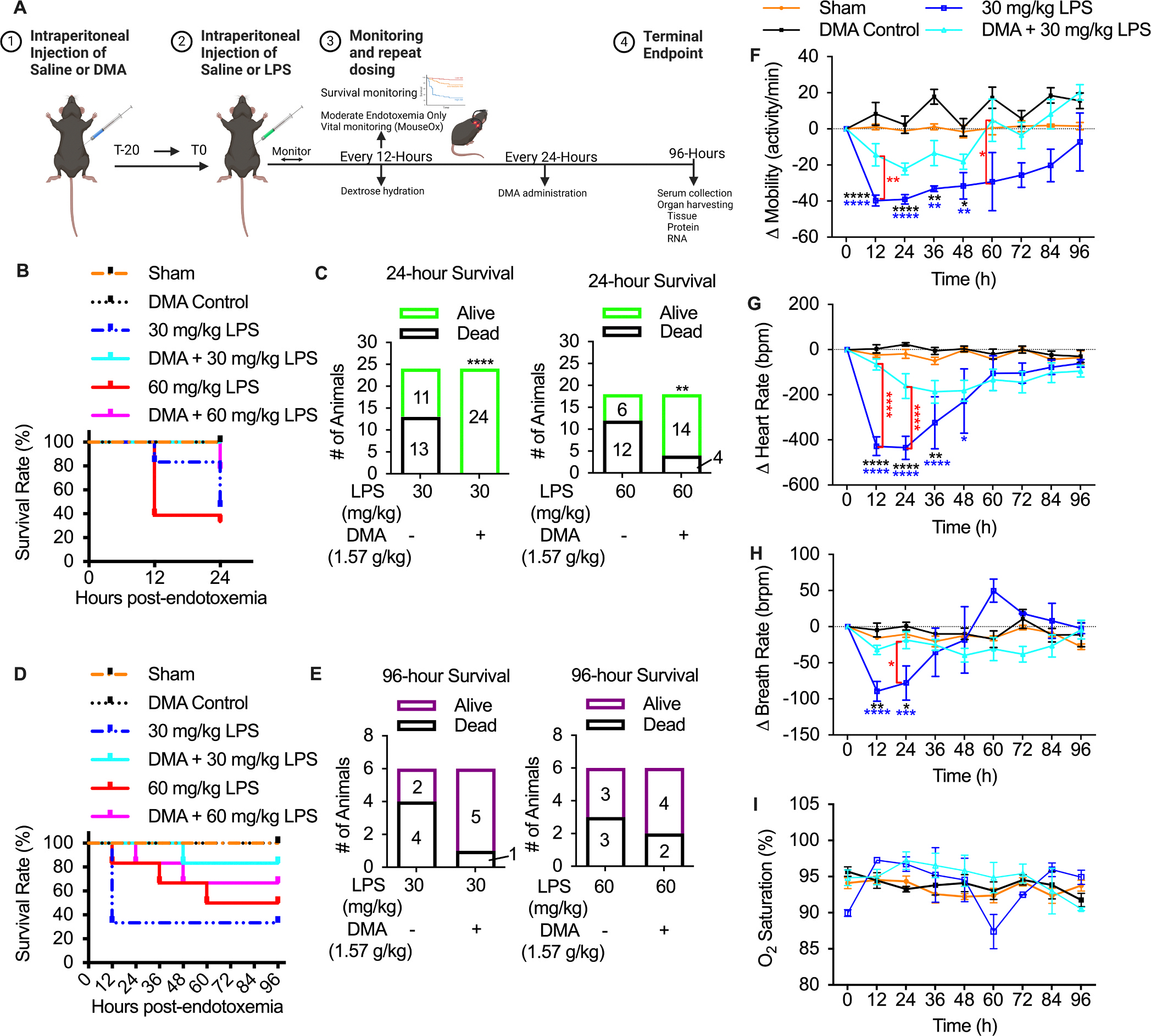
DMA improves survival and stabilizes vital signs during acute and prolonged endotoxemia. **(A)** Experimental timeline and treatment scheme. **(B)** Kaplan–Meier survival curve in acute endotoxemia (24 h) and **(C)** correlation analysis comparing untreated and DMA-treated groups. Moderate and severe endotoxemia were induced using 30 or 60 mg/kg LPS, respectively. *n* = 20 for Sham and DMA control groups; *n* = 24 and *n* = 18 for moderate and severe endotoxemia groups, respectively. **(D)** Kaplan–Meier survival curve in prolonged endotoxemia (96 h) and **(E)** corresponding correlation analysis. *n* = 4 for Sham and DMA control groups; *n* = 6 for endotoxemia groups. **(F–I)** Mouse vital signs were recorded every 12 h via oximetry in prolonged moderate endotoxemia and presented as changes in (F) mobility (ΔMobility), **(G)** heart rate (ΔHeart Rate), **(H)** respiratory rate (ΔBreath Rate), and **(I)** oxygen saturation (O_2_ Sat). *n* = 4 for Sham and DMA control groups; *n* = 6 for moderate endotoxemia groups. Data are presented as mean ± SEM. Correlation analyses were performed using the Chi-square test. Differences in vital signs across timepoints were analyzed using two-way ANOVA with Tukey’s multiple-comparisons test. *P < 0.05, **P < 0.01, ***P < 0.001, ****P < 0.0001.

The overall experimental scheme is shown in Fig. 1A. To determine whether DMA alleviates endotoxemia-induced acute (24-h) and prolonged (96-h) mortality, survival data across 24-, 48-, 72-, and 96-h timepoints were analyzed by Kaplan–Meier survival curves (Fig. 1B, D). Moderate and severe endotoxemia were induced using 30 or 60 mg/kg LPS, respectively, representing distinct levels of disease severity characterized by differential inflammatory responses and mortality. DMA treatment fully prevented mortality in acute moderate endotoxemia (*P* < 0.0001) and reduced mortality to 22.2% in severe endotoxemia (*P* < 0.01) (Fig. 1C). Kaplan–Meier analysis of prolonged endotoxemia revealed survival rates of 33.3% and 50.0% in moderate and severe disease, respectively (Fig. 1D–E). DMA modestly but consistently improved long-term survival to 83.3% in moderate endotoxemia and 66.7% in severe endotoxemia.

To evaluate physiological outcomes in prolonged moderate endotoxemia, body weight (BW) and vital signs—mobility, heart rate (HR), breath rate (BrR), and oxygen saturation—were monitored using MouseOx. BW declined in endotoxemic animals (Supp. Fig. 1 A). The kinetics of ΔMobility, ΔHR, ΔBrR and oxygen saturation are presented in Fig. 1F–I and DMA’s effect on mobility in acute severe endotoxemia is captured in the video. In both Sham and DMA-alone control groups, vital parameters remained stable throughout the observation period. In contrast, endotoxemia markedly reduced ΔMobility (Fig. 1F and video) and ΔHR (Fig. 1G) at T12, T24, T36, and T48, and significantly decreased ΔBrR at T12 and T24 (Fig. 1H) relative to baseline (T0). DMA pretreatment significantly ameliorated these endotoxemia-induced declines, improving ΔMobility at T12 and T60, ΔHR at T12 and T24, and ΔBrR at T24, restoring values to levels not statistically different from the Sham group. Oxygen saturation remained above 85% in all groups, with no significant between-group differences, although the lowest values consistently occurred in endotoxemic mice (Fig. 1I).

### 3.2. DMA pretreatment alleviates systemic and hepatic inflammation in acute endotoxemia

**Fig. 2.**
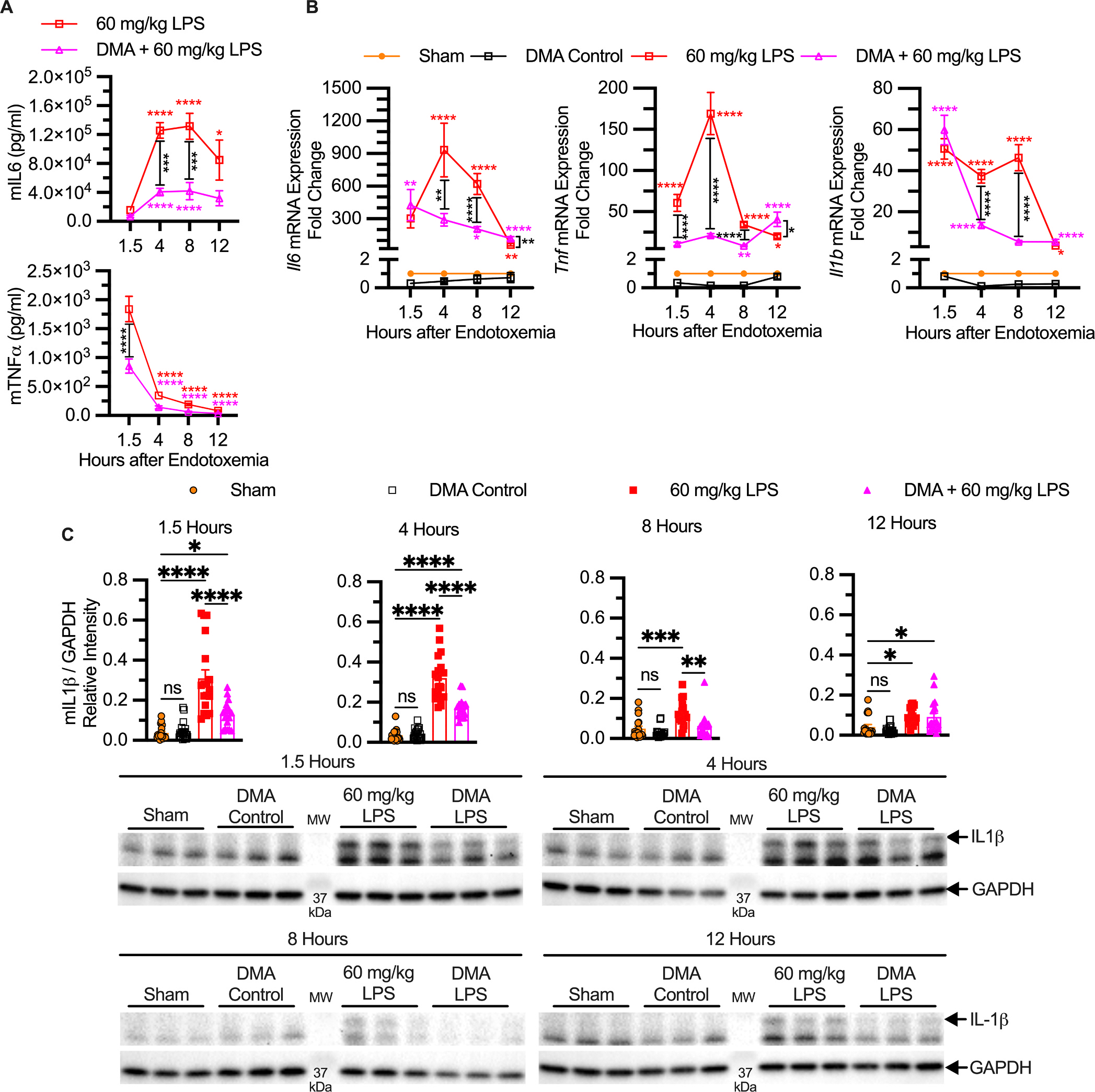
DMA alleviates systemic and hepatic inflammation in acute endotoxemia. (A) Serum concentrations of mIL-6 and mTNF-α in mice with acute endotoxemia. (B) Hepatic mRNA expression of pro-inflammatory cytokine genes (Il6, Tnf, and Il1b) in acute endotoxemia. (C) Densitometric quantification of pro–mIL-1β protein in liver tissue, with representative immunoblots shown below. *n* = 6 per group per time point. Data are presented as mean ± SEM. Serum cytokine levels over time were analyzed by two-way ANOVA with Tukey’s multiple-comparisons test. Liver cytokine mRNA expression at each time point and Western blot densitometry were analyzed by one-way ANOVA with Tukey’s multiple-comparisons test. *P < 0.05, **P < 0.01, ***P < 0.001, ****P < 0.0001.

Because sepsis is driven by dysregulated host immune responses, we first evaluated systemic inflammation by quantifying circulating cytokines. Serum mIL-6 and mTNF-α in Sham and DMA control animals were < 80 pg/ml and below the limit of detection (data not shown). Severe endotoxemia markedly increased serum mIL-6 and mTNF-α, whereas DMA treatment significantly attenuated these elevations—reducing mIL-6 levels by 68% at T4 (*P* < 0.001) and T8 (*P* < 0.001) and mTNF-α by 54% at T1.5 (*P* < 0.0001) (Fig. 2A).

The liver, a central immunomodulatory organ, plays a major role in the systemic inflammatory response in sepsis. To assess hepatic inflammation, we measured expression of cytokine and acute-phase protein (APP)–encoding genes. Pro-inflammatory cytokine transcripts *Il6*, *Tnf* and *Il1b* were first examined (Fig. 2B). DMA alone did not alter expression of these genes. Severe endotoxemia induced profound increases in *Il6* (304-fold), *Tnf* (61-fold), and *Il1b* (51-fold) at T1.5. *Il6* and *Tnf* expression peaked at T4 and then declined at T8 and T12, whereas *Il1b* expression peaked earlier at T1.5 and remained relatively stable until T8 before sharply decreasing at T12. DMA pretreatment significantly suppressed *Il6*, *Tnf* and *Il1b* expression in endotoxemic animals at T4 and T8 (with *Il1b* suppression evident from T4 to T8). Because IL-1β is synthesized as an inactive pro-protein requiring caspase-1–mediated cleavage, we also assessed hepatic pro-mIL-1β protein levels (Fig. 2C). Consistent with transcript data, pro-mIL-1β markedly increased in endotoxemic livers, and DMA pretreatment reduced levels to ~50% of untreated endotoxemia animals from T1.5 through T8.

**Fig. 3.**
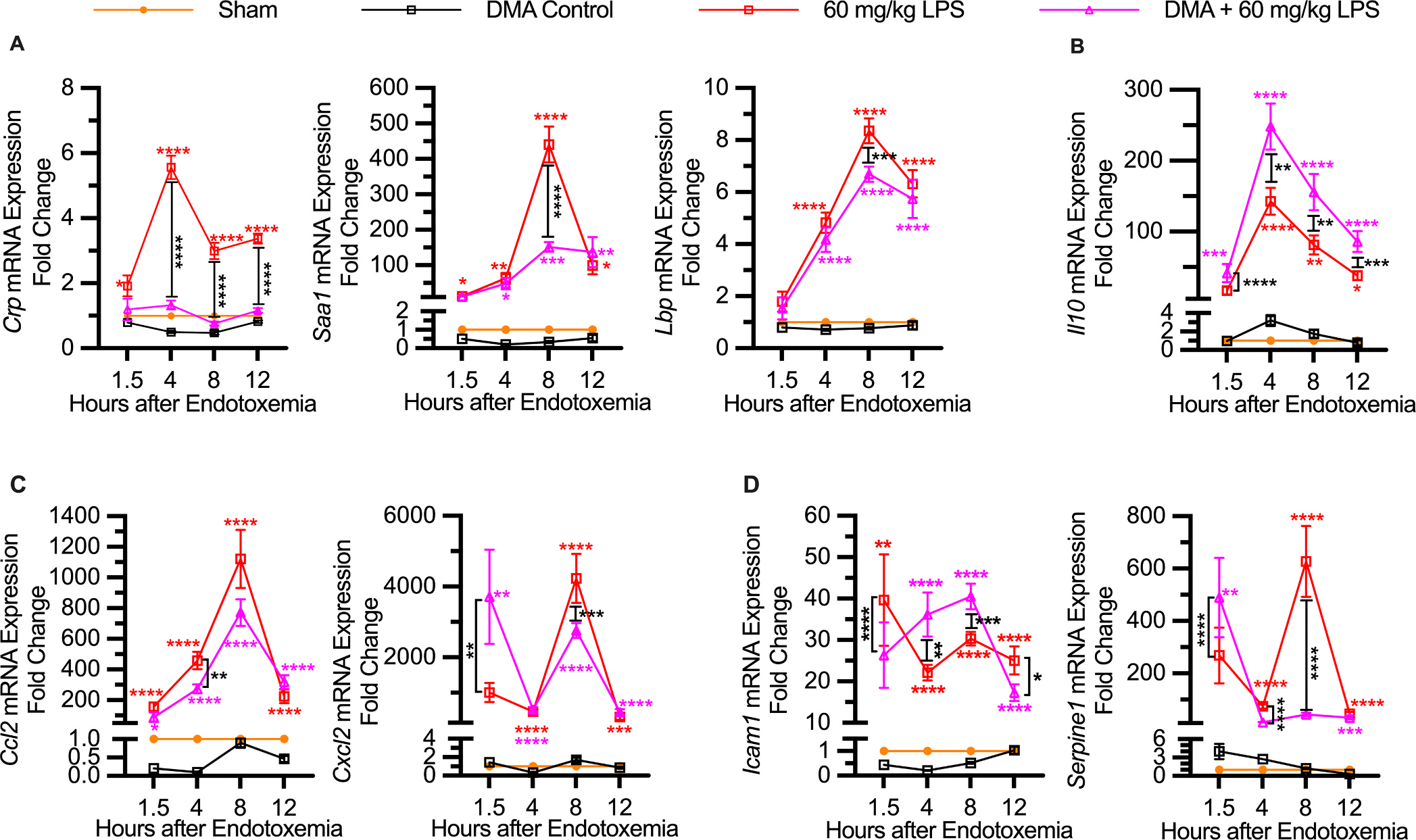
DMA attenuates acute liver inflammation by dampening acute-phase responses, increasing IL-10, and priming the hepatic microenvironment for leukocyte migration. mRNA expression of **(A)** acute-phase proteins *Crp*, *Saa1*, and *Lbp*; **(B)** anti-inflammatory cytokine *Il10*; **(C)** chemokines *Ccl2* and *Cxcl2*; and **(D)** mediators of liver microenvironment remodeling (*Icam1* and *Serpine1*). *n* = 6 per group per time point. Data are presented as mean ± SEM. Statistical analysis of liver cytokine and acute-phase protein mRNA expression at each time point was performed using one-way ANOVA with Tukey’s multiple-comparisons test. *P < 0.05, **P < 0.01, ***P < 0.001, ****P < 0.0001.

We next quantified hepatic APP-encoding genes *Crp*, *Saa1* and *Lbp* (Fig. 3A). DMA alone did not significantly alter expression of *Crp*, *Saa1* or *Lbp*. Endotoxemia induced rapid upregulation of *Crp* and *Saa1* at T1.5, and *Lbp* at T4. *Crp* expression further increased at T4 and remained elevated to T12; *Saa1* and *Lbp* continued increasing to T8 before declining at T12. Remarkably, *Crp* expression in DMA-pretreated animals remained at Sham (basal) levels throughout. *Saa1* expression was reduced to < 50% of untreated endotoxemia levels at T8 and remained low at T12. *Lbp* expression followed a similar kinetic pattern to untreated endotoxemia but was significantly attenuated at T8.

Finally, we assessed the immunoregulatory cytokine *Il10* (Fig. 3B). DMA alone only modestly affected *Il10* expression relative to Sham. In untreated endotoxemia, *Il10* peaked at T4 and gradually declined to T12. In contrast, DMA pretreatment further augmented *Il10* expression at all timepoints relative to untreated endotoxemia animals, suggesting enhanced induction of counter-regulatory immunomodulation.

### 3.3. DMA pretreatment regulates leukocyte migration–associated gene expression in mouse liver during acute severe endotoxemia

Following LPS challenge, the liver undergoes marked cellular and extracellular changes that prime the hepatic microenvironment for leukocyte infiltration. To characterize these alterations, we examined three groups of genes involved in leukocyte recruitment, cell adhesion, and extracellular matrix remodeling. We first analyzed expression of the chemokine transcripts *Ccl2* and *Cxcl2* during acute severe endotoxemia. Endotoxemia induced a robust increase in both chemokines, and DMA pretreatment significantly attenuated this induction at 4 h and 8 h, respectively (Fig. 3C).

We next assessed transcriptional activation of the adhesion molecule *Icam1* and the matrix remodeling factor *Serpine1* (Fig. 3D). While LPS markedly upregulated both genes, DMA pretreatment did not primarily reduce maximal expression levels but rather altered the temporal dynamics of their induction. In controls, peak *Icam1* expression occurred at 1.5 h post-LPS, whereas in DMA-pretreated mice the peak shifted to 8 h. Conversely, *Serpine1* expression peaked at 8 h in the endotoxemia group, but reached its maximal level at 1.5 h in DMA-treated animals. These findings suggest that DMA modulates the kinetics of hepatic leukocyte infiltration programs, rather than simply suppressing their overall magnitude.

### 3.4. DMA pretreatment attenuates activation of NLRP3 inflammasome signaling in mouse liver during acute endotoxemia

**Fig. 4.**
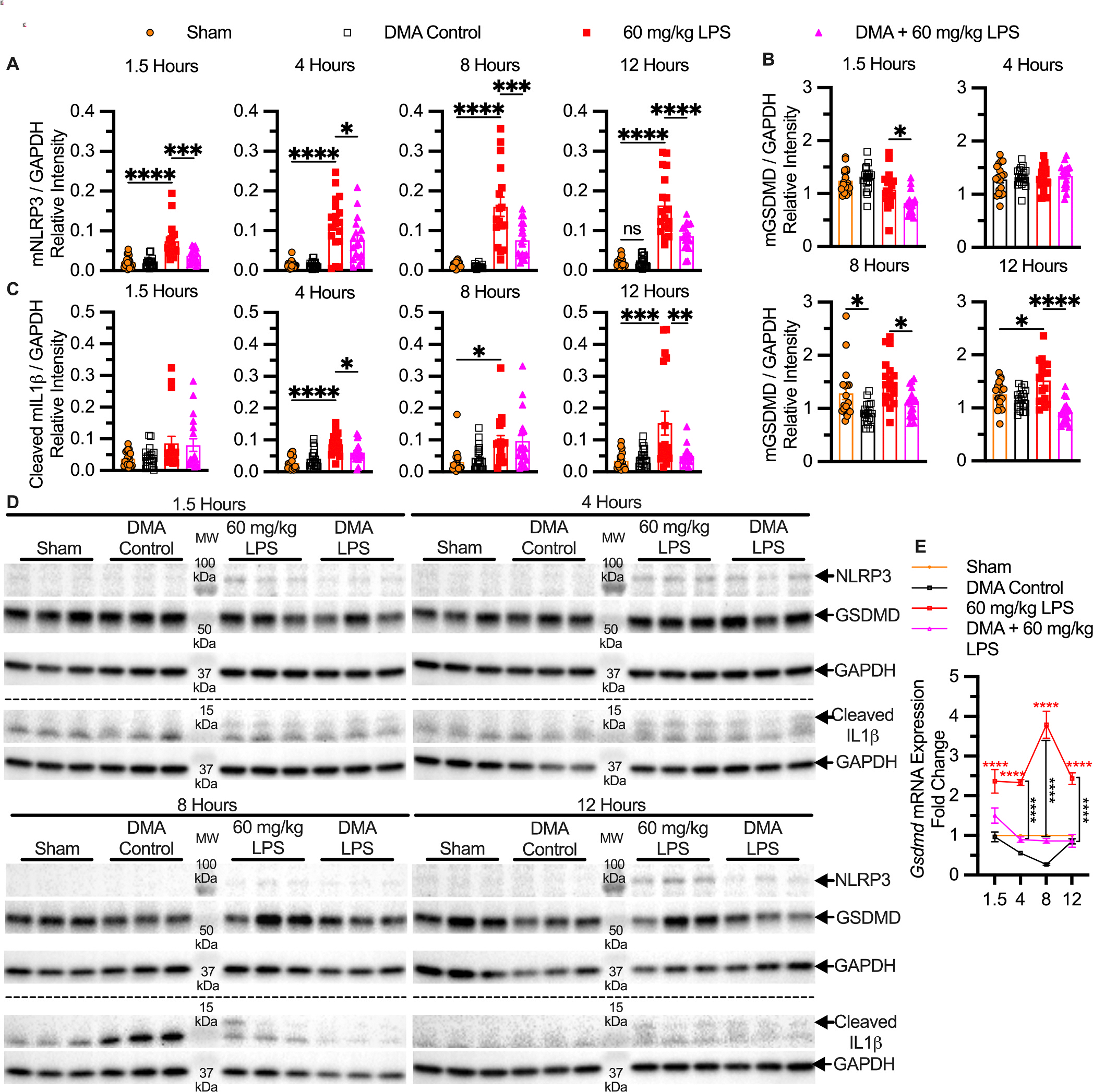
DMA attenuates NLRP3 inflammasome activation and **and IL-1β maturation** in mouse liver during acute endotoxemia. Densitometric quantification of **(A)** mNLRP3, **(B)** mGSDMD, and **(C)** cleaved mIL-1β protein levels in mouse liver. **(D)** Representative Western blots. **(E)** Hepatic *Gsdmd* mRNA expression. *n* = 6 per group per time point. Data are shown as mean ± SEM. Statistical analyses for Western blot and mRNA expression were performed using one-way ANOVA with Tukey’s multiple comparisons test. *P < 0.05, **P < 0.01, ***P < 0.001, ****P < 0.0001.

Given that DMA reduced both systemic and hepatic inflammation in LPS-induced endotoxemia, we next evaluated liver injury and activation of the NLRP3 inflammasome pyroptosis pathway. Severe endotoxemia caused a rapid induction of hepatic NLRP3, with significantly elevated protein expression evident at 1.5 h and sustained through 12 h post-LPS (Fig. 4A, D). Apoptosis-associated speck like protein containing a CARD (ASC) expression was also significantly increased at 12 h (Fig. S2D, E). Despite this induction of NLRP3 and ASC, caspase-1 and cleaved caspase-1 protein levels remained largely unchanged during the acute phase (Supp. Fig. 2A–E). Similarly, cleaved GSDMD, a canonical executor of pyroptotic cell death, was not detected at any time point examined, suggesting that full execution of pyroptosis may be limited under these acute conditions.

Total GSDMD protein increased at 12 h (Fig. 4B, D), whereas cleaved GSDMD was not detectable at any time point (data not shown). Consistent with protein trends, *Gsdmd* transcript expression was significantly increased at all timepoints during acute endotoxemia (Fig. 4E). Cleaved mIL-1β, a downstream readout of inflammasome activation and cytokine maturation, was elevated from 4 to 12 h in severe endotoxemia; this increase was significantly attenuated in DMA pretreated mice at 4 h (P < 0.05) and 12 h (P < 0.01) (Fig. 4C, D).

DMA pretreatment markedly reduced inflammasome signaling in endotoxemic livers. NLRP3 induction was significantly blunted across the acute time window (Fig. 4A, D), and reduced levels of ASC (Fig. S2D, E), GSDMD (Fig. 4B, D), and cleaved IL-1β (Fig. 4C, D) were observed in DMA-pretreated mice at 12 h (and at 4 h for cleaved IL-1β). Moreover, *Gsdmd* transcript levels were restored to baseline in DMA-treated endotoxemia animals (Fig. 4E). Together, these findings indicate that DMA pretreatment suppresses NLRP3 inflammasome signaling and IL-1β maturation in the liver during acute endotoxemia.

### 3.5. DMA regulates leukocyte migration– and infiltration–associated gene expression in mouse liver during prolonged endotoxemia

At 96 h post-LPS (T96), endotoxemia animals exhibited splenomegaly, with significant increases in spleen weight and spleen-to-body-weight ratio in moderate endotoxemia (Fig. S1). Based on these findings, we assessed systemic and hepatic inflammation in prolonged endotoxemia. Serum mIL-6 remained detectable across all endotoxemia groups (Fig. S3A), whereas mTNF-α was below the detection limit (data not shown). In severe endotoxemia, serum mIL-6 significantly declined relative to T12, indicating partial resolution of systemic inflammation. DMA pretreatment mildly reduced serum mIL-6 at T96 in severe endotoxemia animals.

**Fig. 5.**
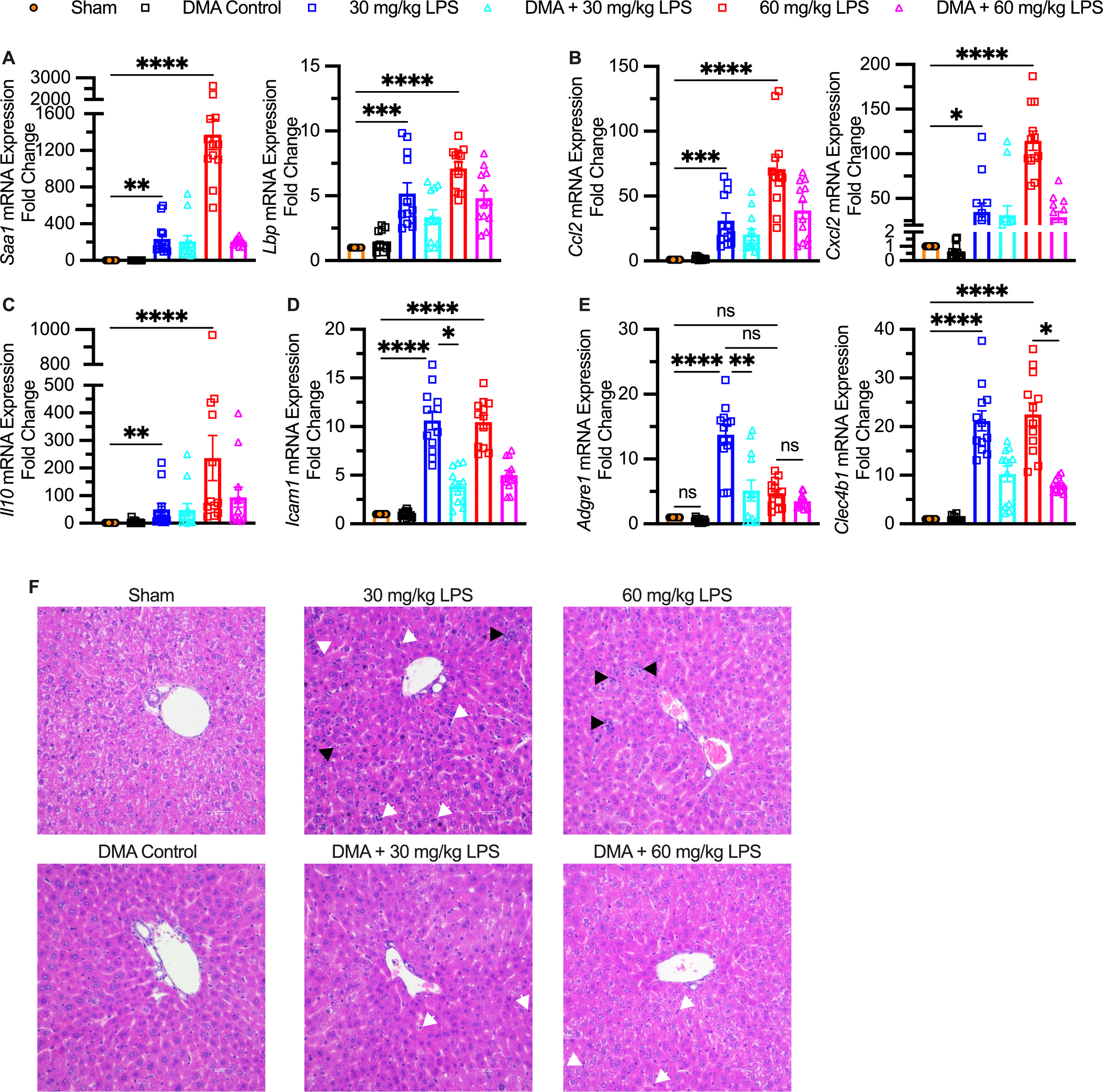
DMA modulates hepatic inflammatory responses during prolonged endotoxemia. Relative mRNA expression of **(A)** acute-phase proteins *Saa1* and *Lbp*; **(B)** chemokines *Ccl2* and *Cxcl2*; **(C)** anti-inflammatory cytokine *Il10*; **(D)** adhesion molecule *Icam1*; and **(E)** leukocyte markers *F4/80 (Adgre1)* and *Clec4b1* in mouse liver. Data are presented as mean ± SEM. Liver mRNA expression at each time point was analyzed by one-way ANOVA with Tukey’s multiple comparisons test. *P < 0.05, **P < 0.01, ***P < 0.001, ****P < 0.0001. **(F)** Representative H&E-stained liver section (400 ×) showing leukocyte infiltration (white arrowhead) and inflammatory foci (black arrowhead). Scale bar = 50 μm. *n* = 4 for Sham and DMA control groups; *n* = 6 for each endotoxemia group.

Despite partial systemic recovery, hepatic inflammation persisted. Transcripts encoding proinflammatory cytokines (*Il6, Tnf, Il1b*; Fig. S3B) and acute-phase proteins (*Saa1, Lbp*; Fig. 5A) remained elevated at T96. In untreated severe endotoxemia, *Il6* and *Il1b* were lower than their peak levels during the acute phase (Fig. 2B). Whereas DMA pretreatment substantially dampened cytokine induction during acute endotoxemia, its suppressive effect was limited at T96. Anti-inflammatory *Il10* expression was significantly elevated in all endotoxemia groups at T96 and increased further in the severe endotoxemia group compared to T12 (Fig. 5C). Together, these transcriptional profiles suggest that endotoxemia-induced liver inflammation had entered a resolution phase by T96.

To examine leukocyte recruitment status, we next analyzed chemokine (*Ccl2, Cxcl2*), adhesion molecule (*Icam1*), and leukocyte marker (*Adgre1*/F4/80, *Clec4b1*) transcripts. *Ccl2* and *Cxcl2* remained elevated at T96, with DMA pretreatment only modestly reducing their expression in severe endotoxemia (Fig. 5B). Notably, *Saa1*, which also acts as a chemoattractant, continued to rise in severe endotoxemia at T96 relative to T12 (Fig. 5A). *Icam1* and *Serpine1* remained elevated in all endotoxemia groups (Fig. 5D, Fig. S3C), with DMA pretreatment significantly reducing *Icam1* in moderate endotoxemia (P < 0.05) and mildly suppressing it in severe endotoxemia, while only marginally affecting *Serpine1*. Long-term endotoxemia also drove significant increases in *Adgre1* and *Clec4b1*, consistent with macrophage and myeloid cell enrichment in liver tissue (Fig. 5E). DMA pretreatment significantly attenuated *Adgre1* expression in moderate endotoxemia (P < 0.01) and *Clec4b1* in severe endotoxemia (P < 0.05).

Finally, liver histology confirmed leukocyte infiltration patterns (Fig. 5F). Minimal infiltrates were detected in Sham and DMA-only controls. In endotoxemia animals, we observed scattered neutrophils (white arrows) and clusters of leukocytes forming inflammatory foci (black arrows). DMA treatment reduced leukocyte infiltration in the livers of endotoxemic mice, consistent with the transcriptional changes observed.

### 3.6. DMA pretreatment attenuates LPS-induced cellular stress in mouse liver during prolonged moderate and severe endotoxemia

**Fig. 6.**
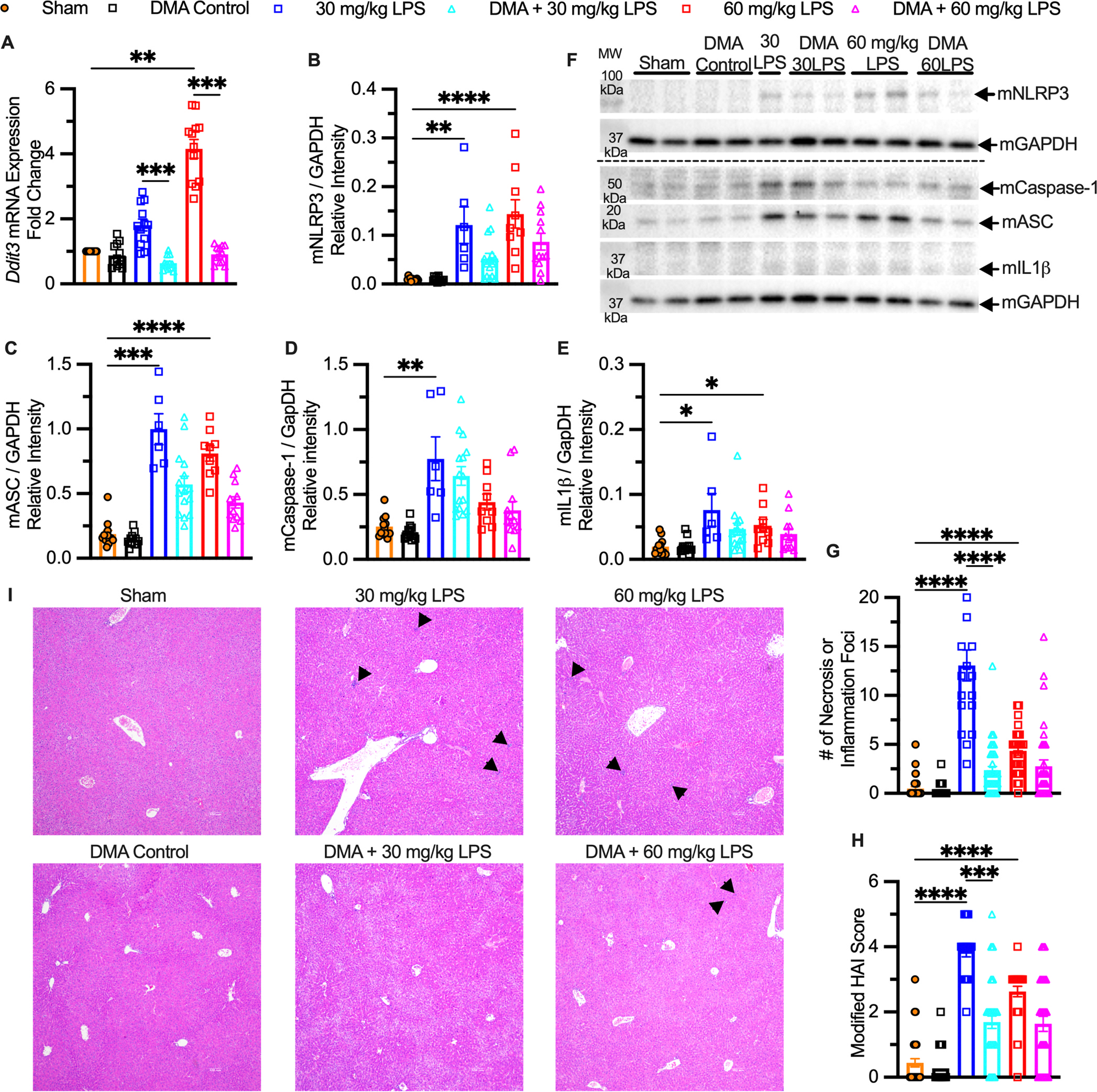
DMA attenuates hepatic inflammation and tissue injury during prolonged endotoxemia. **(A)** Hepatic expression of the cellular stress gene *Ddit3* (CHOP). Densitometric quantification of **(B)** mNLRP3, **(C)** mASC, **(D)** mCaspase-1 pro-protein, and **(E)** mIL-1β pro-protein. Representative western blot images are shown in **(F)**. Data are presented as mean ± SEM. Western blot statistical analysis was performed by one-way ANOVA with Tukey’s multiple comparisons test. Hepatic inflammation severity was assessed by **(G)** quantification of necrotic or inflammatory foci and **(H)** Modified Hepatic Activity Index (HAI) score. Statistical analysis of histological outcomes was performed using the nonparametric Kruskal-Wallis test with Dunn’s post-test. *P < 0.05, **P < 0.01, ***P < 0.001, ****P < 0.0001. **(I)** Representative H&E-stained liver sections at 100 × magnification; inflammatory foci indicated by black arrowheads. Scale bar = 100 μm. *n* = 4 for Sham and DMA-only groups; *n* = 6 for each endotoxemia-treated group.

Given DMA’s effect on leukocyte-associated gene expression in prolonged endotoxemia, we next evaluated inflammation-induced liver injury under these conditions. Although liver weight did not differ among groups, the liver-to-BW ratio was modestly increased in both the moderate endotoxemia and DMA-pretreated groups (Fig. S1C). To assess hepatocellular stress, we measured hepatic mRNA expression of *Ddit3* (CHOP), a marker of ER stress–induced apoptosis. *Ddit3* expression was significantly upregulated in both moderate and severe endotoxemia and was restored to basal levels by DMA pretreatment in both settings (Fig. 6A).

We next examined activation of the NLRP3 inflammasome–mediated pyroptosis pathway. Hepatic mNLRP3 and mASC protein levels were markedly elevated at T96 in mice with moderate and severe endotoxemia (Fig. 6B, C). Notably, mNLRP3 expression in severe endotoxemia remained elevated at levels comparable to T12, whereas mASC expression continued to rise at T96, indicating sustained inflammasome activation. Although not statistically significant, DMA pretreatment reduced hepatic mNLRP3 and mASC levels in both endotoxemia models at T96 (Fig. 6B, C). Levels of mCaspase-1 pro-protein increased in moderate prolonged endotoxemia, an effect that was slightly but not significantly attenuated by DMA (Fig. 6D, F). Likewise, mIL-1β pro-protein levels increased in both moderate and severe prolonged endotoxemia and were modestly mitigated by DMA pretreatment (Fig. 6E, F). Cleaved mCaspase-1 levels remained unchanged across groups (Fig. S3E), and although *Gsdmd* mRNA expression increased in moderate endotoxemia, this did not translate into detectable protein-level changes (Supp. Fig. 3D, F).

Histopathological assessment revealed an increased number of necrotic and inflammatory foci in livers from mice with prolonged moderate or severe endotoxemia (Fig. 6G), indicated by black arrowheads in Fig. 6I. These morphological changes corresponded with significantly elevated modified hepatic activity index (HAI) scores (Fig. 6H). DMA pretreatment markedly reduced both necrotic/inflammatory foci and modified HAI scores in prolonged moderate endotoxemia.

### 3.7. DMA pretreatment suppresses proinflammatory cytokine secretion in leukocytes

**Fig. 7.**
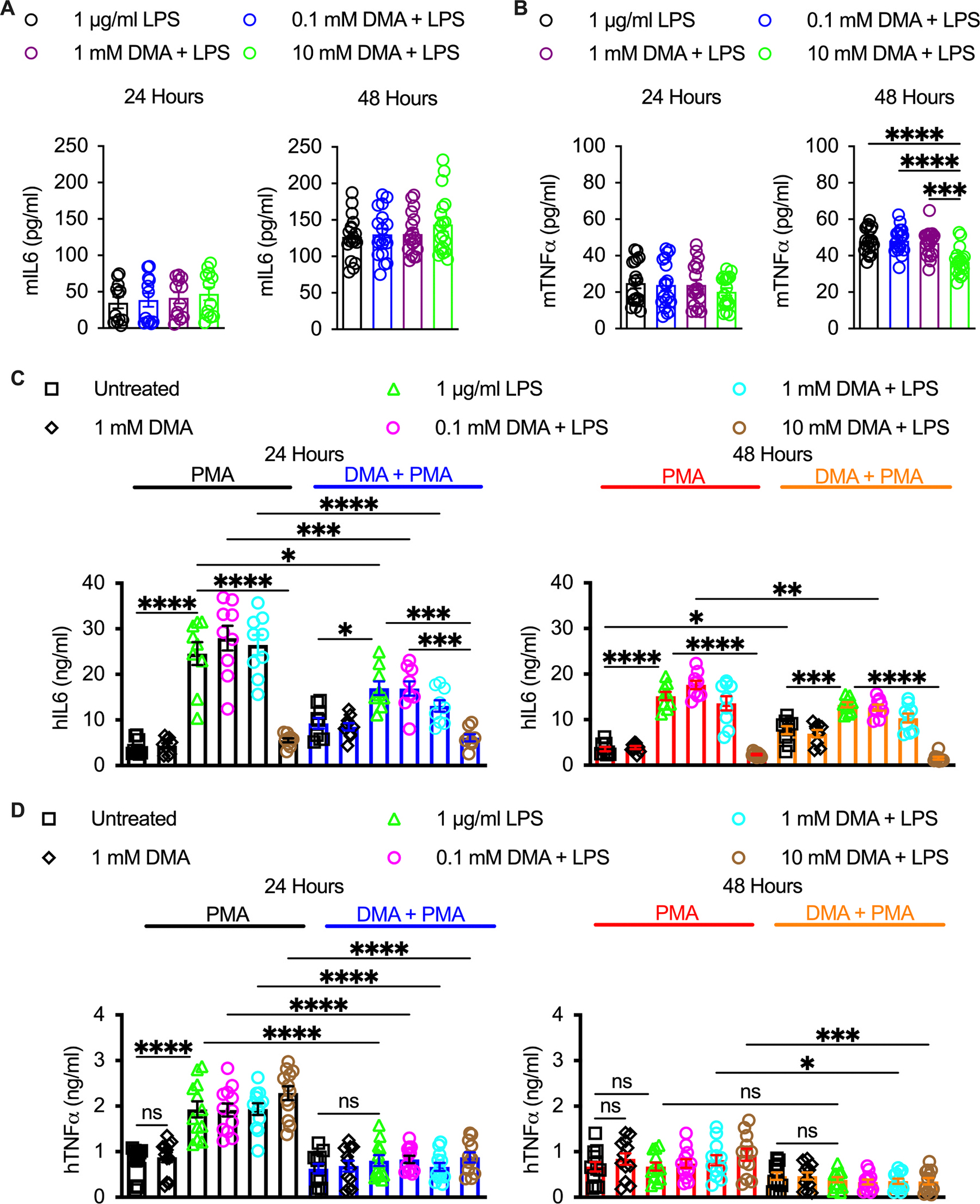
DMA suppresses proinflammatory cytokine secretion in leukocytes. Primary mouse neutrophils were isolated from bone marrow and treated with 1 μg/ml LPS in the presence of increasing concentrations of DMA for 24 or 48 h. Levels of murine IL-6 **(A)** and TNF-α **(B)** in the culture supernatants were quantified by ELISA. Human U937 monocytes were differentiated into macrophages in the absence or presence of DMA and subsequently treated with LPS and increasing concentrations of DMA. Levels of secreted human IL-6 **(C)** and TNF-α **(D)** were measured by ELISA. Data are presented as mean ± SEM. Statistical significance was determined using one-way ANOVA followed by Tukey’s multiple comparisons test. *P < 0.05, **P < 0.01, ***P < 0.001, ****P < 0.0001.

Primary mouse neutrophils and human macrophages were used to examine whether DMA pretreatment modulates leukocyte inflammatory activity. Three noncytotoxic concentrations of DMA (0.1, 1.0, and 10 mM) were selected based on cytotoxicity assays (Supp. Fig. 4). In primary neutrophils, cytokines were below the limit of detection in untreated controls and cells exposed to DMA alone (data not shown). LPS stimulation induced time-dependent secretion of mIL-6 and mTNF-α (Fig. 7A, B). While DMA did not prevent LPS-induced mIL-6 secretion, 10 mM DMA significantly reduced mTNF-α levels at 48 h (P < 0.0001) (Fig. 7B).

To further investigate DMA’s immunomodulatory effects, human U937 monocytes were differentiated into macrophages using PMA in the absence or presence of 10 mM DMA. U937 macrophages differentiated without DMA exhibited robust induction of IL-6 and TNF-α following LPS stimulation (Fig. 7C, D). Differentiation in the presence of DMA (10 mM) significantly reduced hIL-6 secretion at 24 h and 48 h but did not alter hTNF-α secretion (Fig. 7C, D).

Because DMA was administered systemically *prior* to LPS challenge in our murine endotoxemia model, we next evaluated whether exposure to DMA during macrophage differentiation alters subsequent responsiveness to inflammatory stimuli. U937 monocytes were differentiated in the *presence* of DMA (i.e., DMA + PMA groups) and then subjected to the same LPS and DMA treatments. LPS still induced IL-6 secretion in these cells, but levels were markedly lower than in macrophages differentiated without DMA (Fig. 7C). Notably, TNF-α secretion was not increased in cells differentiated in the presence of DMA (Fig. 7D). Moreover, treatment with 10 mM DMA further suppressed IL-6 secretion at 24 h (P < .001) and 48 h (P < 0.0001) in LPS-stimulated U937 cells that were differentiated with DMA (Fig. 7C).

## 4. Discussion and conclusion

Because endotoxemia models isolate the host inflammatory response in the absence of active infection, they are particularly useful for evaluating anti-inflammatory mechanisms. Current sepsis treatment primarily targets pathogen eradication and hemodynamic stabilization, including antimicrobial therapy [18,19], source control interventions [20], fluid resuscitation [21], and vasoactive agents [22]. Adjunctive use of glucocorticoids and corticosteroids aims to limit excessive inflammatory responses [22–24], highlighting the continued need for therapies that directly modulate dysregulated inflammation.

In this study, DMA markedly decreased endotoxemia-induced mortality. The use of two LPS doses enabled us to evaluate DMA efficacy across a spectrum of disease severity, demonstrating protective effects in both moderate and severe endotoxemia. DMA completely eliminated acute mortality in moderate endotoxemia and reduced mortality in severe endotoxemia by approximately 45%. In prolonged endotoxemia, DMA significantly improved long-term survival and reversed early reductions in heart rate, respiratory rate, and mobility. Importantly, these protective effects coincided temporally with the period during which most mortality occurred (<60 h), suggesting that DMA effectively dampened early pathogenic processes that drive lethality.

DMA also substantially attenuated systemic inflammation. Endotoxemia induces robust increases in circulating proinflammatory cytokines, including IL6 [25,26], IL1β [25], and TNFα [25,27]. In untreated animals, serum TNFα peaked rapidly at 1.5 h and declined by 4 h, whereas IL6 increased over the first 8 h and decreased by 12 h, reflecting a self-limiting inflammatory response. DMA significantly reduced peak systemic inflammation, decreasing serum IL6 and TNFα levels by 68% and 54%, respectively. These findings indicate that DMA actively suppresses the magnitude of the cytokine response rather than merely accelerating its resolution.

Given the central role of the liver in coordinating systemic inflammation [28,29], we examined hepatic inflammatory signaling as a potential target of DMA. LPS strongly induced hepatic expression of *Il6*, *Tnf*, and *Il1b* during acute severe endotoxemia, all of which were significantly reduced by DMA. At 96 h, cytokine expression remained elevated but was substantially lower than during the acute phase (Fig. S3), consistent with inflammatory resolution. DMA further decreased these residual inflammatory signals, indicating sustained suppression of hepatic cytokine production.

Because IL1β requires inflammasome-dependent cleavage for activation, we examined hepatic levels of pro- and mature IL1β as well as components of the NLRP3 inflammasome. Both pro- and active IL1β were elevated during acute endotoxemia and were reduced by DMA in parallel with transcript-level changes. Consistent with prior reports of inflammasome activation in sepsis [30], NLRP3 and ASC protein levels were increased in the liver. However, cleaved caspase-1 was not detectably altered (data not shown), suggesting partial inflammasome engagement. DMA-mediated suppression of upstream inflammatory cues may therefore limit IL1β activation independently of caspase-1 cleavage.

DMA also inhibited the hepatic acute-phase response. Acute-phase proteins such as C-reactive protein (CRP), serum amyloid A1/2 (SAA1/2), and LPS-binding protein (LBP) are key amplifiers of inflammation [31–33]. CRP, a liver-derived protein widely used as a clinical biomarker of sepsis severity [34], was induced sixfold by LPS during acute endotoxemia and returned to baseline by 96 h. DMA completely normalized Crp expression during the acute phase. Because CRP promotes tissue factor expression on mononuclear and endothelial cells [35, 36], its suppression by DMA may reduce procoagulant signaling and leukocyte recruitment within the liver.

SAA1/2, which enhances inflammation through induction of cytokines [37], activation of TLR2/4, and engagement of the NLRP3 inflammasome [38], was profoundly induced by LPS. Saa1 expression increased ~450-fold at 8 h and ~1375-fold at 96 h in severe endotoxemia, indicating sustained inflammatory amplification. DMA reduced Saa1 expression by 85% during prolonged severe endotoxemia, suggesting that DMA disrupts both early and persistent SAA-driven inflammatory loops. Notably, while we observed robust modulation of inflammasome-associated proteins, canonical markers of pyroptotic execution (cleaved caspase-1 and cleaved GSDMD) were not detected at the time points examined, suggesting that DMA primarily modulates inflammasome signaling rather than overt pyroptotic cell death under these conditions.

LBP facilitates LPS presentation to the TLR4/CD14 complex, thereby amplifying NF-κB–dependent inflammatory signaling [39]. Hepatic *Lbp* expression was significantly increased during endotoxemia and was suppressed by DMA at both acute (20% at 8 h) and prolonged (32% at 96 h) time points. By limiting LBP availability, DMA may reduce LPS sensing by Kupffer cells and macrophages, contributing to attenuated systemic and hepatic inflammation.

Because proinflammatory cytokines such as IL6, IL1β, and TNFα regulate hepatic acute-phase protein synthesis [31,40], DMA-mediated suppression of these cytokines likely contributes indirectly to reduced *Crp*, *Saa1*, and *Lbp* expression. In parallel, DMA enhanced expression of the anti-inflammatory cytokine IL10. IL10 promotes immune resolution by suppressing proinflammatory cytokine production [41,42], inducing soluble TNF receptors and IL1 receptor antagonists [41], improving survival in sepsis models [43], and protecting the liver from inflammatory and apoptotic injury [44]. DMA further increased LPS-induced Il10 expression during both acute and prolonged endotoxemia, suggesting that DMA shifts the inflammatory balance toward a sustained anti-inflammatory state.

DMA also modulated signals governing leukocyte recruitment and tissue remodeling. Endotoxemia rapidly increased hepatic expression of chemokines (*Ccl2*, Cxcl2) and adhesion molecules (*Icam1*), reflecting early remodeling of the liver microenvironment. *Serpine1* (PAI-1), a regulator of extracellular matrix remodeling and endothelial function [45–47], exhibited a transient spike during acute endotoxemia and remained elevated during prolonged disease. DMA reduced chemokine expression at multiple time points and altered the kinetics of *Icam1* and *Serpine1* expression, consistent with reduced leukocyte adhesion and infiltration. At 96 h, DMA modestly suppressed residual expression of these factors.

SAA1/2 further contributes to immune cell recruitment and extracellular matrix degradation by acting as a chemoattractant and inducing matrix metalloproteinases [38,48]. Its sustained elevation in severe endotoxemia suggests ongoing chemotaxis and tissue remodeling, both of which were significantly suppressed by DMA. Consistent with this, markers of macrophages (*F4/80*, *Adgre1*) and myeloid cells (*Clec4b*, *Clec4b1*) were reduced by DMA in long-term endotoxemia. DMA also decreased hepatic expression of the cellular stress marker *Ddit3* (GADD153) and reduced leukocyte infiltration and inflammatory foci in liver tissue, indicating broad protection against inflammation-induced tissue injury.

Finally, DMA reduced expression of *Serpine1* at early (4–8 h) and late (96 h) stages of endotoxemia. SERPINE1 inhibits fibrinolysis and is strongly associated with sepsis severity, organ dysfunction, disseminated intravascular coagulation, and mortality in patients [49–53]. LPS-induced *Serpine1* expression in our model suggests impaired fibrinolysis and increased thrombotic risk. DMA-mediated suppression of *Serpine1* may therefore mitigate coagulopathy and endothelial dysfunction, consistent with reports identifying SERPINE1-expressing hepatic endothelial cells as contributors to sepsis-associated liver injury [54].

While these findings are encouraging, several important limitations should be considered when interpreting their translational relevance. First, the LPS-induced endotoxemia model used in this study recapitulates key features of the host inflammatory response but does not fully model infection-driven sepsis, in which ongoing pathogen replication, microbial clearance, and host–pathogen interactions play critical roles. Second, DMA was administered using a prophylactic dosing paradigm at relatively high doses, which may not directly reflect clinically feasible treatment strategies in patients with established sepsis. Third, although the liver is a central immunoregulatory organ and a major source of systemic inflammatory mediators, our mechanistic analyses were largely focused on hepatic responses and do not capture the full spectrum of multi-organ dysfunction that characterizes sepsis. Accordingly, the present study is best interpreted as demonstrating that DMA exerts potent anti-inflammatory and immunomodulatory effects in endotoxemia. Future studies will be required to evaluate the efficacy of DMA in infection-based sepsis models (e.g., cecal ligation and puncture), to test therapeutic (post-insult) dosing paradigms, and to assess its impact on additional organ systems. These investigations will be critical for determining the translational potential of DMA as an adjunctive therapy for sepsis.

In summary, DMA consistently decreased pathological responses to endotoxemia across systemic, hepatic, cellular, and molecular levels. DMA reduced acute mortality, improved long-term survival, suppressed proinflammatory cytokine and acute-phase protein expression, enhanced anti-inflammatory signaling, limited leukocyte infiltration, and attenuated markers of endothelial dysfunction and coagulopathy. In this initial mechanistic study, we employed a preventive dosing paradigm in which DMA was administered prior to the onset of endotoxemia, allowing us to test the hypothesis that DMA modulates early events in the inflammatory cascade that ultimately drive mortality. Because DMA was administered prophylactically, the current data support a preventive effect in endotoxemia. Future studies will evaluate the efficacy of DMA when administered at later time points, after endotoxemia is established, to better reflect its translational potential as a treatment for sepsis. Although the precise molecular targets of DMA require further investigation, these findings support DMA as a potent modulator of endotoxemia-driven inflammation and a promising candidate for adjunctive therapy in sepsis.

## Supplementary Material

MMC1

MMC2

MMC7

MMC5

MMC6

MMC3

MMC4

## Data Availability

Data will be made available on request.

## Appendix A. Supporting information

Supplementary data associated with this article can be found in the online version at doi:10.1016/j.biopha.2026.119403.

## References

[1] Singer M, Deutschman CS, Seymour CW, Shankar-Hari M, Annane D, Bauer M, Bellomo R, Bernard GR, Chiche J-D, Coopersmith CM, Hotchkiss RS, Levy MM, Marshall JC, Martin GS, Opal SM, Rubenfeld GD, van del Poll T, Vincent J-L, Angus DC, The third international consensus definitions for sepsis and septic shock (Sepsis-3), JAMA 315 (8) (2016) 801–810. 26903338 10.1001/jama.2016.0287 PMC4968574

[2] Fay K, Sapiano MRP, Gokhale R, Dantes R, Thompson N, Katz DE, Ray SM, Wilson SE, Perlmutter R, Nadle J, Godine D, Frank L, Brousseau G, Johnston H, Bamberg W, Dumyati G, Nelson D, Lynfield R, DeSilva M, Kainer M, Zhang A, Ocampo V, Samper M, Pierce R, Irizarry L, Sievers M, Maloney M, Fiore A, Magill SS, Epstein L, Assessment of health care exposures and outcomes in adult patients with sepsis and septic shock, JAMA Netw. Open 3 (7) (2020) e206004. 32633762 10.1001/jamanetworkopen.2020.6004 PMC7341174

[3] Hotchkiss RS, Moldawer LL, Opal SM, Reinhart K, Turnbull IR, Vincent J-L, Sepsis and septic shock, Nat. Rev. Dis. Prim. 2 (2016) 16045. 28117397 10.1038/nrdp.2016.45 PMC5538252

[4] White LE, Hassoun HT, Bihorac A, Moore LJ, Sailors RM, McKinley BA, Valdivia A, Moore FA, Acute kidney injury is surprisingly common and a powerful predictor of mortality in surgical sepsis, J. Trauma Acute Care Surg. 75 (3) (2013) 432–438. 24089113 10.1097/TA.0b013e31829de6cd PMC3823059

[5] Howell MD, Davis AM, Management of sepsis and septic shock, JAMA 317 (8) (2017) 847–848. 28114603 10.1001/jama.2017.0131

[6] Polat G, Ugan RA, Cadirci E, Halici Z, Sepsis and septic shock: current treatment strategies and new approaches, Eurasia J. Med. 49 (1) (2017) 53–58. 10.5152/eurasianjmed.2017.17062 PMC5389495 28416934

[7] Hempel G, Oechtering D, Lanvers-Kaminsky C, Klingebiel T, Vormoor J, Gruhn B, Boss J, Cytotoxicity of dimethylacetamide and pharmacokinetics in children receiving intravenous busulfan, J. Clin. Oncol. 25 (13) (2007) 1772–1778. 17470868 10.1200/JCO.2006.08.8807

[8] Andersson BS, Madden T, Tran HT, Hu WW, Blume KG, Chow DS-L, Champlin RE, Vaughan WP, Acute safety and pharmacokinetics of intravenous busulfan when used with oral busulfan and cyclophosphamide as pretransplantation conditioning therapy: a phase I study, Biol. Blood Marrow Transpl. 6 (5a) (2000) 548–554. 10.1016/s1083-8791(00)70064-4 11071260

[9] Bhagwatwar HP, Phadungpojna S, Chow DSL, Andersson BS, Formulation and stability of busulfan for intravenous administration in high-dose chemotherapy, Cancer Chemother. Pharm. 37 (5) (1996) 401–408. 10.1007/s002800050404 8599861

[10] Sundaram S, Ashby CR Jr., Pekson R, Sampat V, Sitapara R, Mantell L, Chen C-H, Yen H, Abhichandani K, Munnangi S, Khadtare N, Stephani RA, Reznik SE, N,N-dimethylacetamide regulates the proinflammatory response associated with endotoxin and prevents preterm birth, Am. J. Pathol. 183 (2) (2013) 422–430. 23770347 10.1016/j.ajpath.2013.05.006 PMC3730776

[11] Ghayor C, Gjoksi B, Dong J, Siegenthaler B, Caflisch A, Weber FE, N. N-dimethylacetamide, a drug excipient that acts as bromodomain ligand for osteoporosis treatment, Sci. Rep. 7 (2017) 42108. 28176838 10.1038/srep42108 PMC5296751

[12] Pekson R, Poltoratsky V, Gorasiya S, Sundaram S, Ashby CR Jr., Vancurova I, Reznik SE, N,N-dimethylacetamide significantly attenuates LPS- and TNFalpha-induced proinflammatory responses via inhibition of the nuclear factor kappa B pathway, Mol. Med. 22 (2016) 747–758. 27782292 10.2119/molmed.2016.00017 PMC5193464

[13] Koya JB, Shen T, Lu G, Gauthier A, Mantell L, Ashby CR Jr., S.E. Reznik, FDA-approved excipient N,N-dimethylacetamide attenuates inflammatory bowel disease in in vitro and in vivo models, Fortune J. Health Sci. 5 (2022) 499–509.

[14] Reznik SE, Kashou A, Ward D, Yellon SM, N,N-dimethylacetamide blocks inflammation-induced preterm birth and remediates maternal systemic immune responses, Sci. Rep. 15 (2025) 8234. 40065144 10.1038/s41598-025-93282-0 PMC11893883

[15] Mir A, Acosta T, Concheiro-Guisan M, Yellon SM, Patel K, Reznik SE, Improving the safety of N,N-dimethylacetamide (DMA) as a potential treatment for preterm birth in a pregnant mouse model using a vaginal nanoformulation, BBA Mol. Basis Dis. 1871 (2025) 167822. 10.1016/j.bbadis.2025.167822 PMC11994577 40174791

[16] Wei ZH, Koya J, Acharekar N, Trejos J, Dong XD, Schanne FA, Ashby CR, Reznik SE, N,N-dimethylacetamide targets neuroinflammation in Alzheimer's disease in in vitro and ex vivo models, Sci. Rep. 13 (1) (2023) 7077. 37127686 10.1038/s41598-023-34355-w PMC10151369

[17] Swamydas M, Luo Y, Dorf ME, Lionakis MS, Isolation of mouse neutrophils, Curr. Protoc. Immunol. 110 (2015) 3.20.1–3.20.15. 10.1002/0471142735.im0320s110 PMC4574512 26237011

[18] Niederman MS, Baron RM, Bouadma L, Calandra T, Daneman N, DeWaele J, Kolle MH, Lipman J, Nair GB, Initial antimicrobial management of sepsis, Crit. Care 25 (1) (2012) 307. 10.1186/s13054-021-03736-w PMC8390082 34446092

[19] Chanderraj R, Admon AJ, He Y, Nuppnau M, Albin OR, Prescott HC, Dickson RP, Sjoding MW, Mortality of patients with sepsis administered piperacillin-tazobactam vs cefepime, JAMA Intern. Med. 184 (7) (2024) 769–777. 38739397 10.1001/jamainternmed.2024.0581 PMC11091820

[20] Reitz KM, Kennedy J, Li SR, Handzel R, Tonetti DA, Neal MD, Zuckerbraun BS, Hall DE, Sperry JL, Angus DC, Tzeng E, Seymour CW, Association between time to source control in sepsis and 90-day mortality, JAMA Surg. 157 (9) (2022) 817–826. 35830181 10.1001/jamasurg.2022.2761 PMC9280613

[21] National Heart, Lung, and Blood Institute Prevention and Early Treatment of Acute Lung Injury Clinical Trials Network, Early restrictive or liberal fluid management for sepsis-induced hypotension, N. Engl. J. Med. 388 (6) (2023) 499–510. 36688507 10.1056/NEJMoa2212663 PMC10685906

[22] Ammar MA, Ammar AA, Wieruszewski PM, Bissell BD, Long MT, Albert L, Khanna AK, Sacha GL, Timing of vasoactive agents and corticosteroid initiation in septic shock, Ann. Intensive Care 12 (1) (2022) 47. 35644899 10.1186/s13613-022-01021-9 PMC9148864

[23] Annane D, Bellissant E, Bollaert P-E, Briegel J, Keh D, Kupfer Y, Pirracchio R, Rochwerg B, Corticosteroids for treating sepsis in children and adults, Cochrane Database Syst. Rev. (2025). 10.1002/14651858.CD002243.pub4 PMC6953403 31808551

[24] Vincent J-L, Current sepsis therapeutics, eBioMedicine 86 (2022) 104318. 36470828 10.1016/j.ebiom.2022.104318 PMC9782815

[25] Terrando N, Fidalgo AR, Vizcaychipi M, Cibelli M, Ma D, Monaco C, Feldmann M, Maze M, The impact of IL-1 modulation on the development of lipopolysaccharide-induced cognitive dysfunction, Crit. Care 14 (3) (2010) R88. 20470406 10.1186/cc9019 PMC2911722

[26] de Azevedo LCP, Park M, Noritomi DT, Marciel AT, Brunialti MK, Salomão R, Characterization of an animal model of severe sepsis associated with respiratory dysfunction, Clin. (Sao Paulo) 62 (4) (2007) 491–498. 10.1590/s1807-59322007000400017 17823713

[27] Alexander JJ, Jacob A, Cunningham P, Hensley L, Quigg RJ, TNF is a key mediator of septic encephalopathy acting through its receptor, TNF receptor-1, Neurochem. Int. 52 (3) (2008) 447–456. 17884256 10.1016/j.neuint.2007.08.006 PMC3191465

[28] Koo DJ, Chaudry IH, Wang P, Kupffer cells are responsible for producing inflammatory cytokines and hepatocellular dysfunction during early sepsis, J. Surg. Res. 83 (2) (1999) 151–157. 10329110 10.1006/jsre.1999.5584

[29] Vollmar B, Rüttinger D, Wanner GA, Leiderer R, Menger MD, Modulation of Kupffer cell activity by gadolinium chloride in endotoxemic rats, Shock 6 (6) (1996) 434–441. 8961394 10.1097/00024382-199612000-00008

[30] Li Z, Liu T, Feng Y, Tong Y, Jia Y, Wang C, Cui R, Qu K, Liu C, Zhang J, PPARgamma alleviates sepsis-induced liver injury by inhibiting hepatocyte pyroptosis via inhibition of the ROS/TXNIP/NLRP3 signaling pathway, Oxid. Med. Cell. Longev. (2022) 1269747. 35136484 10.1155/2022/1269747 PMC8818407

[31] Castell JV, Gómez-Lechón MJ, David M, Andus T, Geiger T, Trullenque R, Fabra R, Heinrich PC, Interleukin-6 is the major regulator of acute phase protein synthesis in adult human hepatocytes, FEBS Lett. 242 (2) (1989) 237–239. 2464504 10.1016/0014-5793(89)80476-4

[32] Castell JV, Gómez-Lechón MJ, David M, Fabra R, Trullenque R, Heinrich PC, Acute-phase response of human hepatocytes: regulation of acute-phase protein synthesis by interleukin-6, Hepatology 12 (5) (1990) 1179–1186. 1699862 10.1002/hep.1840120517

[33] Bas S, Gauthier BR, Spenato U, Stingelin S, Gabay C, CD14 is an acute-phase protein, J. Immunol. 172 (7) (2004) 4470–4479. 15034063 10.4049/jimmunol.172.7.4470

[34] Ostrowski SR, Berg RMG, Windeløv NA, Meyer MA, Plovsing RR, Møller K, Johansson PI, Coagulopathy, catecholamines, and biomarkers of endothelial damage in experimental human endotoxemia and in patients with severe sepsis: a prospective study, J. Crit. Care 28 (0035) (2013) 586–596. 23731819 10.1016/j.jcrc.2013.04.010

[35] Dhainaut J-F, Marin N, Mignon A, Vinsonneau C, Hepatic response to sepsis: interaction between coagulation and inflammatory processes, 7 Suppl, Crit. Care Med. 29 (2001) S42–S47. 11445733 10.1097/00003246-200107001-00016

[36] Cirillo P, Golino P, Calabro P, Calì G, Ragni M, De Rosa S, Cimminò G, Pacileo M, de Palma R, Forte L, Gargiulo A, Corigliano FG, Angri V, Spagnuolo R, Nitsch L, Chiariello M, C-reactive protein induces tissue factor expression and promotes smooth muscle and endothelial cell proliferation, Cardiovasc. Res. 68 (1) (2005) 47–55. 16023093 10.1016/j.cardiores.2005.05.010

[37] Furlaneto CJ, Campa A, A novel function of serum amyloid A: a potent stimulus for the release of tumor necrosis factor-alpha, interleukin-1beta, and interleukin-8 by human blood neutrophil, Biochem. Biophys. Res. Commun. 268 (2) (2000) 405–408. 10679217 10.1006/bbrc.2000.2143

[38] Ye RD, Sun L, Emerging functions of serum amyloid A in inflammation, J. Leukoc. Biol. 98 (6) (2015) 923–929. 26130702 10.1189/jlb.3VMR0315-080R PMC6608020

[39] He Z, Song Z, Meng L, Cheng W, Huang F, Zheng M, Xu W, Xiao R, Fang H, Zhu Y, Lipopolysaccharide-induced transcriptional changes in LBP-deficient rat and its possible implications for liver dysregulation during sepsis, J. Immunol. Res. (2021) 8356645. 35005033 10.1155/2021/8356645 PMC8739918

[40] Bode JG, Albrecht U, Häussinger D, Heinrich PC, Schaper F, Hepatic acute phase proteins—regulation by IL-6- and IL-1-type cytokines involving STAT3 and its crosstalk with NF-kappaB-dependent signaling, Eur. J. Cell Biol. 91 (6–7) (2012) 496–505. 22093287 10.1016/j.ejcb.2011.09.008

[41] Delano MJ, Ward PA, The immune system's role in sepsis progression, resolution, and long-term outcome, Immunol. Rev. 274 (1) (2016) 330–353. 27782333 10.1111/imr.12499 PMC5111634

[42] Knolle P, Schlaak J, Uhrig A, Kempf P, Meyer zum Büschenfelde KH, Gerken G, Human Kupffer cells secrete IL-10 in response to lipopolysaccharide (LPS) challenge, J. Hepatol. 22 (2) (1995) 226–229. 7790711 10.1016/0168-8278(95)80433-1

[43] Emmanuilidis K, Weighardt H, Maier S, Gerauer K, Fleischmann T, Zheng XX, Hancock WW, Holzmann B, Heidecke C-D, Critical role of Kupffer cell-derived IL-10 for host defense in septic peritonitis, J. Immunol. 167 (7) (2001) 3919–3927. 11564810 10.4049/jimmunol.167.7.3919

[44] Zhu R, Guo W, Fang H, Cao S, Yan B, Chen S, Zhang K, Zhang S, Kupffer cell depletion by gadolinium chloride aggravates liver injury after brain death in rats, Mol. Med. Rep. 17 (5) (2018) 6357–6362. 29488608 10.3892/mmr.2018.8646 PMC5928625

[45] Simone TM, Higgins CE, Czekay R-P, Law BK, Higgins SP, Archambeault J, Kutz SM, Higgins PJ, SERPINE1: a molecular switch in the proliferation-migration dichotomy in wound-activated keratinocytes, Adv. Wound Care 3 (3) (2014) 281–290. 10.1089/wound.2013.0512 PMC3955966 24669362

[46] Chen T-Y, Zhou M, Lin M-Q, Liang S-T, Yan Y, Wang S-M, Fang C-S, Li D, Ruan Y, Research progress on the SERPINE1 protein and chronic inflammatory diseases of the upper respiratory tract: a literature review, Int. Arch. Allergy Immunol. 182 (11) (2021) 1097–1102. 33946071 10.1159/000516195

[47] Zhang W-J, Wojta J, Binder BR, Notoginsenoside R1 counteracts endotoxin-induced activation of endothelial cells in vitro and endotoxin-induced lethality in mice in vivo, Arterioscler. Thromb. Vasc. Biol. 17 (3) (1997) 465–474. 9102164 10.1161/01.atv.17.3.465

[48] Hansen MT, Forst B, Cremers N, Quagliata L, Ambartsumian N, Grum-Schwensen B, Klingelhöfer J, Abdul-Al A, Herrmann P, Osterland M, Stein U, Nielsen GH, Scherer PE, Lukanidin E, Sleeman JP, Grigorian M, A link between inflammation and metastasis: serum amyloid A1 and A3 induce metastasis, and are targets of metastasis-inducing S100A4, Oncogene 34 (4) (2015) 424–435. 24469032 10.1038/onc.2013.568

[49] Mavrommatis AC, Theodoridis T, Economou M, Kotanidou A, Al Eli M, Christopoulou-Kokkinou V, Zakynthinos SG, Activation of the fibrinolytic system and utilization of the coagulation inhibitors in sepsis: comparison with severe sepsis and septic shock, Intensive Care Med 27 (2001) 1853–1859. 11797019 10.1007/s00134-001-1139-8

[50] Green J, Doughty L, Kaplan SS, Sasser H, Carcillo JA, The tissue factor and plasminogen activator inhibitor type-1 response in pediatric sepsis-induced multiple organ failure, Thromb. Haemost. 87 (2) (2002) 218–223. 11858480

[51] Paulus P, Jennewein C, Zacharowski K, Biomarkers of endothelial dysfunction: can they help us deciphering systemic inflammation and sepsis? Biomarkers 16 (Suppl. 1) (2011) S11–S21. 21707440 10.3109/1354750X.2011.587893

[52] Yang K-Y, Liu K-T, Chen Y-C, Chen C-S, Lee Y-C, Perng R-P, Feng J-Y, Plasma soluble vascular endothelial growth factor receptor-1 levels predict outcomes of pneumonia-related septic shock patients: a prospective observational study, Crit. Care 15 (1) (2011) R11. 21219633 10.1186/cc9412 PMC3222041

[53] Hermans PWM, Hibberd ML, Booy R, Daramola O, Hazelzet JA, de Groot R, Levin M, 4G/5G promoter polymorphism in the plasminogen-activator-inhibitor-1 gene and outcome of meningococcal disease, Lancet 354 (9178) (1999) 556–560. 10470700 10.1016/s0140-6736(99)02220-5

[54] Chen G, Ren C, Xiao Y, Wang Y, Yao R, Wang Q, You G, Lu M, Yan S, Zhang X, Zhang J, Yao Y, Zhou H, Time-resolved single-cell transcriptomics reveals the landscape and dynamics of hepatic cells in sepsis-induced acute liver dysfunction, JHEP Rep. 5 (6) (2023) 100718. 37122356 10.1016/j.jhepr.2023.100718 PMC10130477

