# Supplementary material for: The FDA-approved excipient N,N-dimethylacetamide improves survival and attenuates inflammatory pathways in a murine model of endotoxemia
Source: Biomed Pharmacother. Author manuscript; Available in PMC 2026 Jul 6. (PMC13334508; doi:10.1016/j.biopha.2026.119403)
Supplement: MMC1 [file NIHMS2181270-supplement-MMC1.docx]

| Primary Antibody | Vendor | Catalog # | Molecular Weight | Dilution |
| --- | --- | --- | --- | --- |
| Cleaved IL-1b | Cell Signaling | 63124 | 17 kDa | 1:1000 |
| Pro-IL1b | Abcam | 234437 | 30 kDa | 1:1000 |
| Cleaved GSDMD | Cell Signaling | 10137 | 31 kDa | 1:1000 |
| GSDMD | Cell Signaling | 39754 | 53 kDa | 1:1000 |
| Cleaved Caspase-1 | Abcam | 89332 | 22 kDa | 1:1000 |
| Pro-Caspase-1 | Cell Signaling | 179515 | 45, 42, 35 kDa | 1:1000 |
| ASC | Cell Signaling | 67824 | 22 kDa | 1:1000 |
| NLRP3 | Abcam | 270449 | 118 kDa | 1:1000 |
| GAPDH | Cell Signaling | 2118 | 37 kDa | 1:2500 |

**Table S1. Primary antibodies used for western blotting.**
