# Supplementary material for: The FDA-approved excipient N,N-dimethylacetamide improves survival and attenuates inflammatory pathways in a murine model of endotoxemia
Source: Biomed Pharmacother. Author manuscript; Available in PMC 2026 Jul 6. (PMC13334508; doi:10.1016/j.biopha.2026.119403)
Supplement: MMC2 [file NIHMS2181270-supplement-MMC2.docx]

| **Targets** | **5’ – Sequence – 3’** |
| --- | --- |
| mIL6 f | TACCACTTCACAAGTCGGAGGC |
| mIL6 r | CTGCAAGTGCATCATCGTTGTTC |
| mTNFa f | GGCAGGTCTACTTTGGAGTCATTGC |
| mTNFa r | ACATTCGAGGCTCCAGTGAATTCGG |
| mIL1β f | TGGACCTTCCAGGATGAGGACA |
| mIL1β r | GTTCATCTCGGAGCCTGTAGTG |
| mCCL2 f | AGGTCCCTGTCATGCTTCTG |
| mCCL2r | TCTGGACCCATTCCTTCTTG |
| mIL-10 f | CGGGAAGACAATAACTGCACCC |
| mIL-10 r | CGGTTAGCAGTATGTTGTCCAGC |
| mGSDMD f | GGTGCTTGACTCTGGAGAACTG |
| mGSDMD r | GCTGCTTTGACAGCACCGTTGT |
| mCRP f | GATTCCTGAGGCTCCAACACAC |
| mCRP r | ACAGTGTAGCCCTTGTGCAGAC |
| mLBP f | TCCATCGGTGTCCGAGGCAAAT |
| mLBP r | AGGTCCACTGAAATGGTGACACC |
| F4/80 f | CGTGTTGTTGGTGGCACTGTGA |
| F4/80 r | CCACATCAGTGTTCCAGGAGAC |
| mClec4b f | CAAGAGTGAGGAGAACTGCTCC |
| mClec4b r | GCAGCATGAATGTCCAAGATCCC |
| mICAM-1 f | AAACCAGACCCTGGAACTGCAC |
| mICAM-1 r | GCCTGGCATTTCAGAGTCTGCT |
| mGADD153 f | GGAGGTCCTGTCCTCAGATGAA |
| mGADD153 r | GCTCCTCTGTCAGCCAAGCTAG |
| SSA1/2 f | CTCCTATTAGCTCAGTAGGTTGTG |
| SSA1/2 r | CACTTCCAAGTTTCTGTTTATTACCC |
| mSERPINE1 f | CCTCTTCCACAAGTCTGATGGC |
| mSERPINE1 r | GCAGTTCCACAACGTCATACTCG |
| mClu f | GATGATCCACCAGGCTCAACAG |
| mClu r | ACACAGTGCGGTCATCTTCACC |
| mCYP2e1 f | AGGCTGTCAAGGAGGTGCTACT |
| mCYP2e1 r | AAAACCTCCGCACGTCCTTCCA |
| mGapDH f | CATCACTGCCACCCAGAAGACTG |
| mGapDH r | ATGCCAGTGAGCTTCCCGTTCAG |

**Table S2. Primers used for qPCR**
