# Supplementary figures and images for: The FDA-approved excipient N,N-dimethylacetamide improves survival and attenuates inflammatory pathways in a murine model of endotoxemia
Source: Biomed Pharmacother. Author manuscript; Available in PMC 2026 Jul 6. (PMC13334508; doi:10.1016/j.biopha.2026.119403)

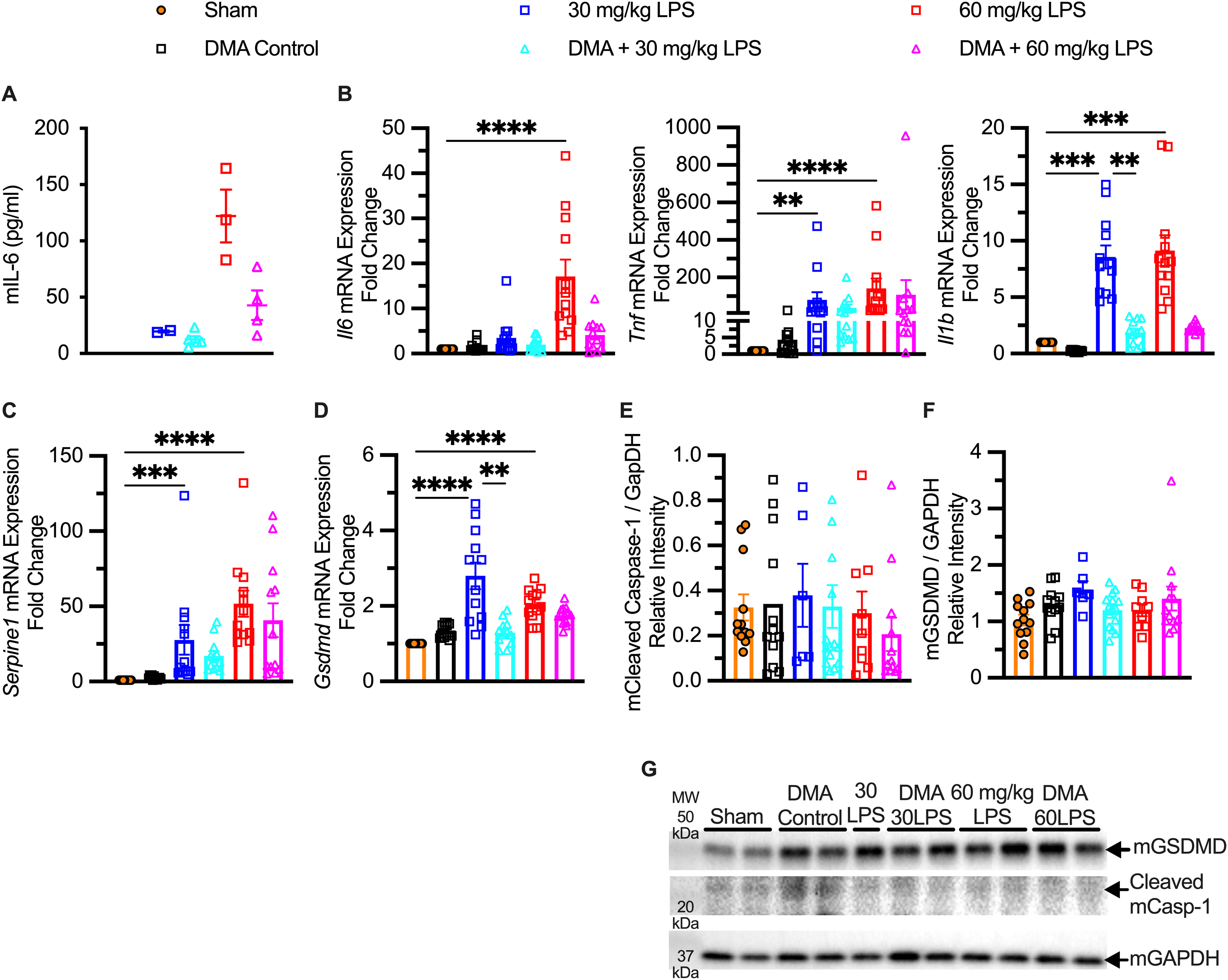

Supplement: MMC5 [file NIHMS2181270-supplement-MMC5.jpg]

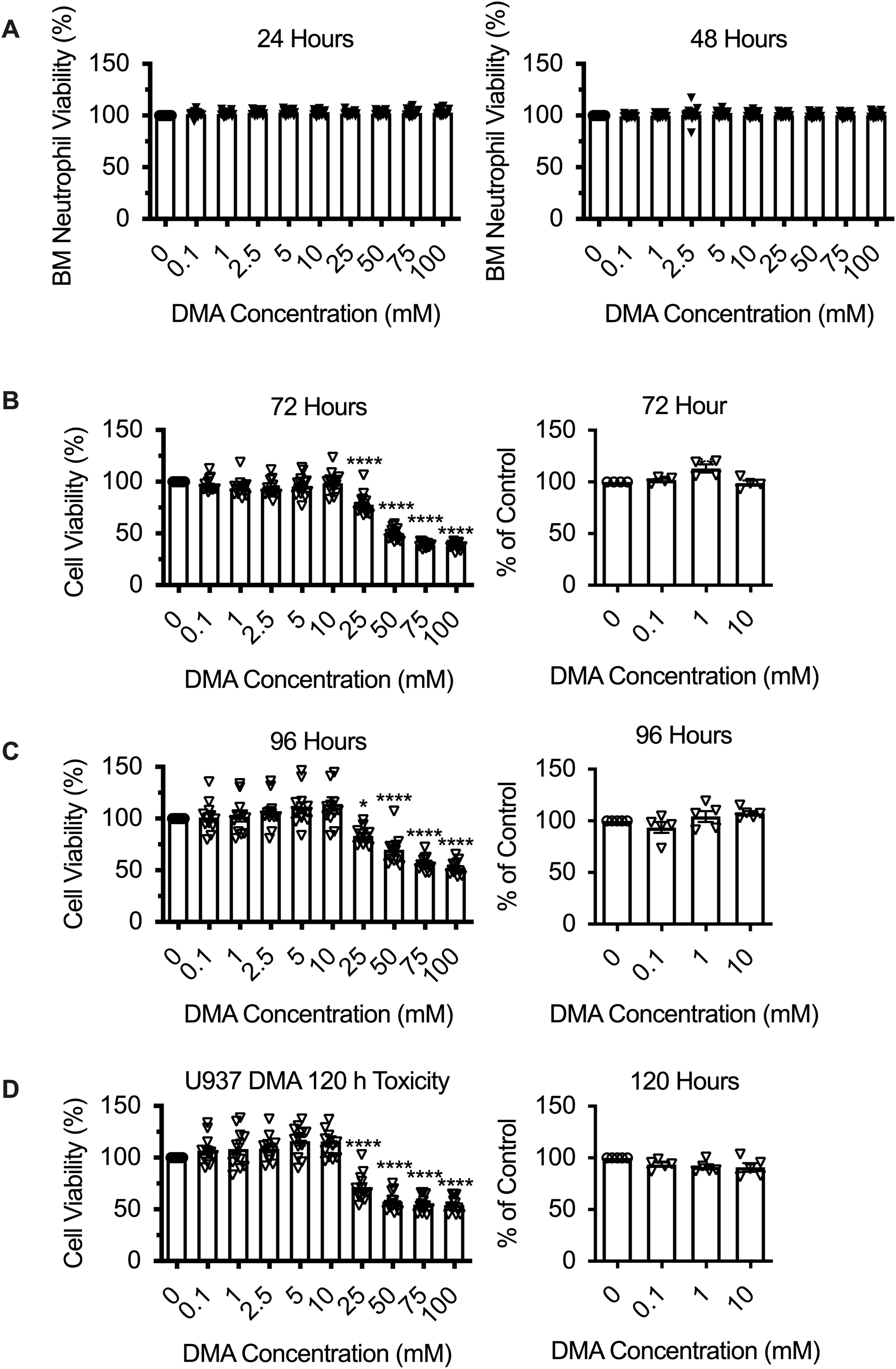

Supplement: MMC6 [file NIHMS2181270-supplement-MMC6.jpg]

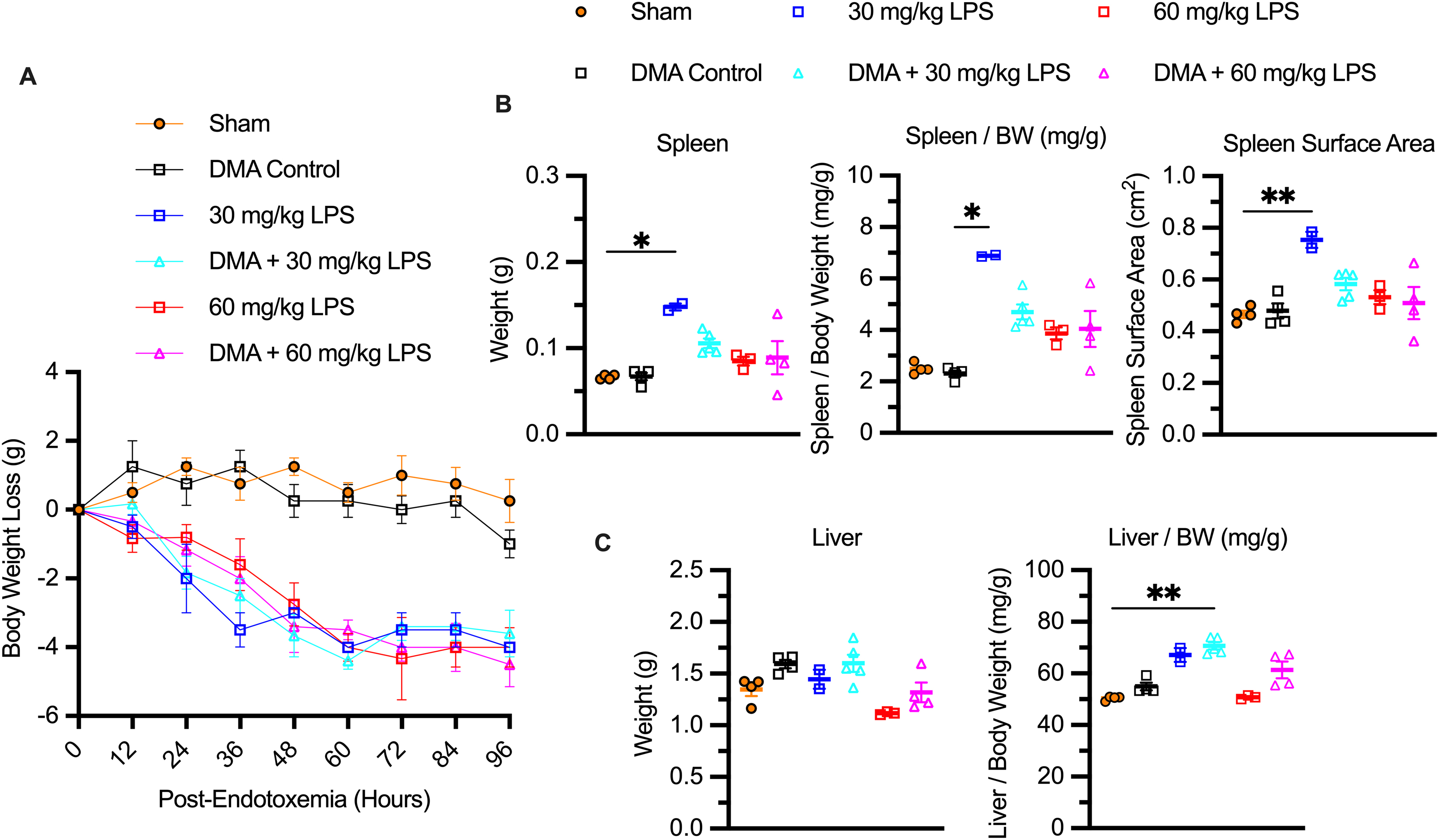

Supplement: MMC3 [file NIHMS2181270-supplement-MMC3.jpg]

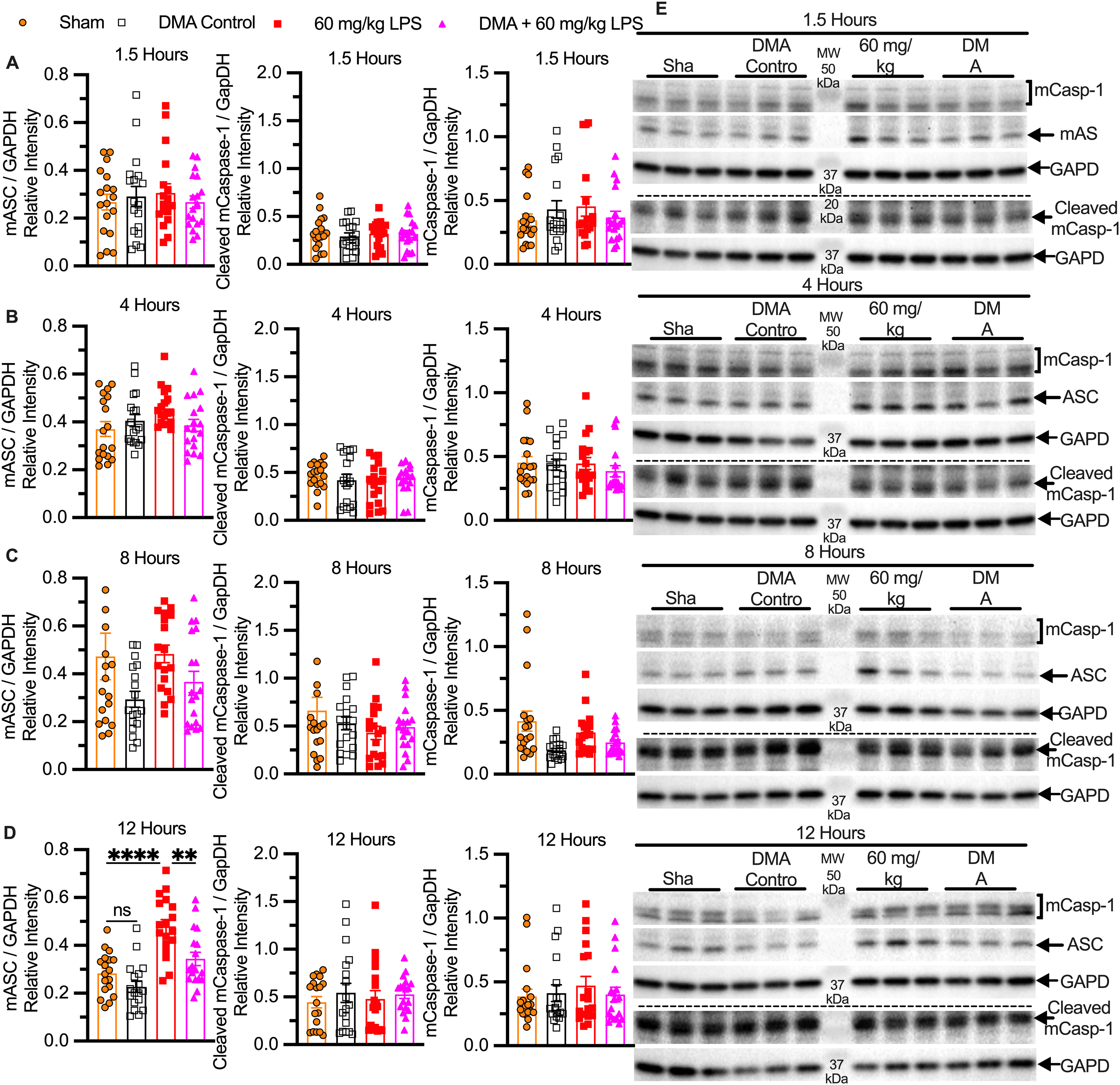

Supplement: MMC4 [file NIHMS2181270-supplement-MMC4.jpg]
